# Optimal methods of vitamin D supplementation to prevent acute respiratory infections: a systematic review, dose–response and pairwise meta-analysis of randomized controlled trials

**DOI:** 10.1186/s12937-024-00990-w

**Published:** 2024-08-14

**Authors:** Chih-Hung Wang, Lorenzo Porta, Ting-Kai Yang, Yu-Hsiang Wang, Tsung-Hung Wu, Frank Qian, Yin-Yi Han, Wang-Huei Sheng, Shyr-Chyr Chen, Chien-Chang Lee, Shan-Chwen Chang

**Affiliations:** 1 Department of Emergency Medicine, College of Medicine, National Taiwan University, Taipei, Taiwan; 2 Department of Emergency Medicine, Zhongzheng Dist, National Taiwan University Hospital, No.7, Zhongshan S. Rd, Taipei City 100, Taiwan, ROC; 3 Department of Emergency Medicine, ASST Grande Ospedale Metropolitano Niguarda, Milan, Italy; 4 School of Medicine and Surgery, Department of Emergency Medicine, Università Degli Studi Di Milano Bicocca, Milan, Italy; 5 College of Medicine, National Taiwan University, Taipei, Taiwan; 6 Sections of Cardiovascular Medicine, Department of Medicine, Boston University, Avedisian School of Medicine, Chobanian &, Boston, MA USA; 7 Department of Trauma, National Taiwan University Hospital, Taipei, Taiwan; 8 Department of Internal Medicine, National Taiwan University Hospital, Taipei, Taiwan; 9 Department of Information Management, Ministry of Health and Welfare, Taipei, Taiwan

**Keywords:** Vitamin D, Acute respiratory infection, Seasonal effects, Dosage, Meta-analysis, Dose–response analysis

## Abstract

**Background:**

Vitamin D supplementation may prevent acute respiratory infections (ARIs). This study aimed to identify the optimal methods of vitamin D supplementation.

**Methods:**

PubMed, Embase, Cochrane Central Register of Controlled Trials, Web of Science, and the ClinicalTrials.gov registry were searched from database inception through July 13, 2023. Randomized-controlled trials (RCTs) were included. Data were pooled using random-effects model. The primary outcome was the proportion of participants with one or more ARIs.

**Results:**

The analysis included 43 RCTs with 49320 participants. Forty RCTs were considered to be at low risk for bias. The main pairwise meta-analysis indicated there were no significant preventive effects of vitamin D supplementation against ARIs (risk ratio [RR]: 0.99, 95% confidence interval [CI]: 0.97 to 1.01, *I*^*2*^ = 49.6%). The subgroup dose–response meta-analysis indicated that the optimal vitamin D supplementation doses ranged between 400–1200 IU/day for both summer-sparing and winter-dominant subgroups. The subgroup pairwise meta-analysis also revealed significant preventive effects of vitamin D supplementation in subgroups of daily dosing (RR: 0.92, 95% CI: 0.85 to 0.99, *I*^*2*^ = 55.7%, number needed to treat [NNT]: 36), trials duration < 4 months (RR: 0.81, 95% CI: 0.67 to 0.97, *I*^*2*^ = 48.8%, NNT: 16), summer-sparing seasons (RR: 0.85, 95% CI: 0.74 to 0.98, *I*^*2*^ = 55.8%, NNT: 26), and winter-dominant seasons (RR: 0.79, 95% CI: 0.71 to 0.89, *I*^*2*^ = 9.7%, NNT: 10).

**Conclusion:**

Vitamin D supplementation may slightly prevent ARIs when taken daily at doses between 400 and 1200 IU/d during spring, autumn, or winter, which should be further examined in future clinical trials.

**Supplementary Information:**

The online version contains supplementary material available at 10.1186/s12937-024-00990-w.

## Background

Acute respiratory infections (ARIs) are one of the leading causes of morbidity and mortality worldwide [1, 2], with a substantial economic burden [3]. The incident cases of ARIs reached more than 17 billion in 2019 [1], with an estimated 2.6 million fatalities associated with ARIs [2].

Vitamin D plays a pivotal role in modulating the immune system, affecting both innate and adaptive immunity [4, 5] by maintaining barrier integrity through tight and adherens junctions, which block pathogen entry. It boosts immune proteins like human cathelicidin LL-37 and defensins [4], vital for infection control. For example, when respiratory syncytial virus penetrates lung alveoli, it triggers the vitamin D metabolism pathway, increasing cathelicidin production [6–8], which disrupts pathogens’ membranes and reduces viral load. Additionally, defensins, produced by leukocytes and epithelial cells, attach to influenza virus surfaces [6, 7], lessening their virulence. Through these mechanisms, vitamin D underpins a sophisticated immune defense strategy, orchestrating a multifaceted response against pathogens to prevent ARIs.

Observational studies [9] indicated an independent association between reduced serum levels of 25-hydroxyvitamin D (the primary vitamin D metabolite) and an increased incidence of ARIs. Nevertheless, the meta-analytic results [10–14] of randomized controlled trials (RCTs) were inconsistent regarding the preventive effects of vitamin D supplementation [10–14]. Most recommended vitamin D supplementation doses aim to facilitate musculoskeletal health [15–17]. There is a knowledge gap concerning the optimal methods of vitamin D required to prevent ARIs. Various dosing strategies for vitamin D have been employed in RCTs, leading to significant heterogeneity and inconsistent results in previous meta-analyses [10–14].

In the current study, we conducted a dose–response meta-analysis to identify the optimal doses of vitamin D supplementation. We also performed pair-wise meta-analysis to determine the overall preventive effects of vitamin D. Finally, we performed subgroup analysis to demonstrate the specific setting for vitamin D to most effectively prevent ARIs.

## Materials and methods

We performed this systematic review and meta-analysis following the Preferred Reporting Items for Systematic Reviews and Meta-Analyses (PRISMA) 2020 statement [18] and registered in PROSPERO (CRD42023423693). Institutional review board approval was not required since we used previously published studies.

### Data sources and search strategy

Two investigators (THW and YHW) independently searched PubMed (inception year: 1996), Embase (inception year: 1947), the Cochrane Central Register of Controlled Trials (inception year: 1996), Web of Science (inception year: 2012), and the ClinicalTrials.gov registry (inception year: 2000) from database inception through July 13, 2023. For the literature search, two sets of search terms were set up to represent vitamin D and ARIs [12] (Supplemental Table 1). No restrictions were employed during the literature search. To ensure completeness, we cross-checked the references of relevant review articles, meta-analyses and trials included.

### Study selection

Two investigators (THW and YHW) independently scanned both titles and abstracts of all retrieved articles and selected those pertinent to this review. The following pre-specified inclusion criteria were used: (a) being a double‐blind RCT, (b) comparing different doses of vitamin D supplementation with or without a placebo group, (c) the events of ARI pre-specified and collected prospectively as an efficacy outcome. Studies reporting the long-term follow-up results of the original RCTs were excluded. After retrieving the full reports of potentially relevant trials, two reviewers (THW and YHW) independently assessed each study’s eligibility based on the inclusion and exclusion criteria. Differences of opinion regarding study eligibility were settled by consensus.

### Data extraction and risk of bias assessment

Three investigators (CHW, LP, TKY) independently extracted qualitative and quantitative data, and a fourth investigator (CCL) adjudicated discordant assessments. We extracted the following data: trial information (study site, duration, time of the year involved), patient characteristics (age, sex, baseline 25-hydroxyvitamin D concentration, proportion of vitamin D deficiency, comorbidities), strategies of vitamin D supplementation (dose, administration frequency), and patient outcomes (definitions of ARI, follow-up duration and serious adverse effects). The average daily dose of vitamin D (IU/d) was calculated by dividing the supplementation dose by the entire study period (if vitamin D was administered only once) or the period of the dosing cycle (if vitamin D was administered daily, weekly, or monthly). We contacted the study authors to provide missing data.

The primary outcome was the proportion of participants with one or more ARIs, defined as any events related to upper, lower or unclassified respiratory tract infection.

Three investigators (CHW, LP, TKY) independently assessed the risk of bias of each RCT by the Version 2 of the Cochrane risk-of-bias tool for randomized trials (RoB 2) [19]; any discrepancies were resolved by consensus.

### Statistical analysis

In the main analysis, we first conducted the dose–response meta‐analysis of weighted relative risks (RRs) between different doses of vitamin D supplementation. We adopted a “one‐stage” [20] natural cubic spline regression model based on a random effects model [21]. We used the placebo dose as the reference for all analyses. We pooled all included studies into a continuous dose‐response curve, and then we estimated the preventive effect of vitamin D on the incidence of ARI from the curve at the given doses. Without pre-specifying parameters about the shape of the association, we used restricted cubic splines of vitamin D supplementation doses with 3 knots at fixed percentiles (10%, 50%, and 90%) [22]. Estimates of the parameters were obtained using restricted maximum likelihood [20, 22]. According to the dose–response curve, preventive effects of vitamin D supplementation were estimated at daily doses of 400, 800, and 1200 IU/d, which were pre-specified according to previous studies [15–17].

Subsequently, we performed pairwise DerSimonian and Laird random-effects [21] meta‐analyses of weighted RRs of all studies to obtain the overall effect estimates comparing two dose levels of vitamin D supplementation. We also stratified the comparisons by different comparator groups, including vitamin D supplementation vs control and higher vs lower doses of vitamin D supplementation. For studies comparing two or more vitamin D regimens with the control, we selected the regimen with the highest daily dose for pooling.

In the subgroup analysis, we also conducted both dose-repose and pairwise meta-analyses. The subgroups were stratified based on pre-specified trial-level variables, including mean age at enrolment (< 7, 7–17, 18–65, or > 65 years) (Children above 7 years old were considered school age and therefore used to stratify the age group), male proportion (more or less than 60%), comorbidity (general or disease-specific population), baseline 25-hydroxyvitamin D concentration (greater or less than 50 nmol/L), dosing frequency (bolus once, daily, weekly, or monthly), climatic zone of the study site (tropical, subtropical, or temperate zone), trial duration (< 4 months, 4–12 months, or > 12 months), and study seasons (summer-inclusive vs summer-sparing, and winter-dominant vs winter-non-dominant). Summer-inclusive and -sparing trials were defined as those involving summer or not during the study period, respectively. Winter-dominant trials were defined as those with winter longer than 50% of the study periods, including studies performed during winter, autumn and winter, or winter and spring; the rest were considered winter-non-dominant.

In the sensitivity analyses, we tested the influence of different definitions of ARIs on the preventive effects of vitamin D supplementation. The definitions of ARIs included upper, lower or mixed upper and lower respiratory tract infections, and influenza. We also specifically examined the preventive effects of vitamin D supplementation under three different dosing regimens, including daily, daily or weekly, and bolus or monthly administration.

We examined small-study effects by visualizing funnel plots and performing Egger’s test [23, 24]. The heterogeneity was assessed using the *I*^*2*^ statistic and the Cochran’s Q test of heterogeneity [25, 26]. For meta-analytic results that demonstrated significant preventive effects of vitamin D supplementation, the number needed to treat (NNT) was calculated by taking the inverse of the difference between the control event rate and the experimental event rate. A two-tailed p-value of < 0.05 was considered statistically significant. We used Stata statistical software (Stata Corp, College Station, TX, 2019) for our data analysis, including the one‐stage approach based on the drmeta command [27].

## Results

### Study inclusion process and characteristics

**Fig. 1:**
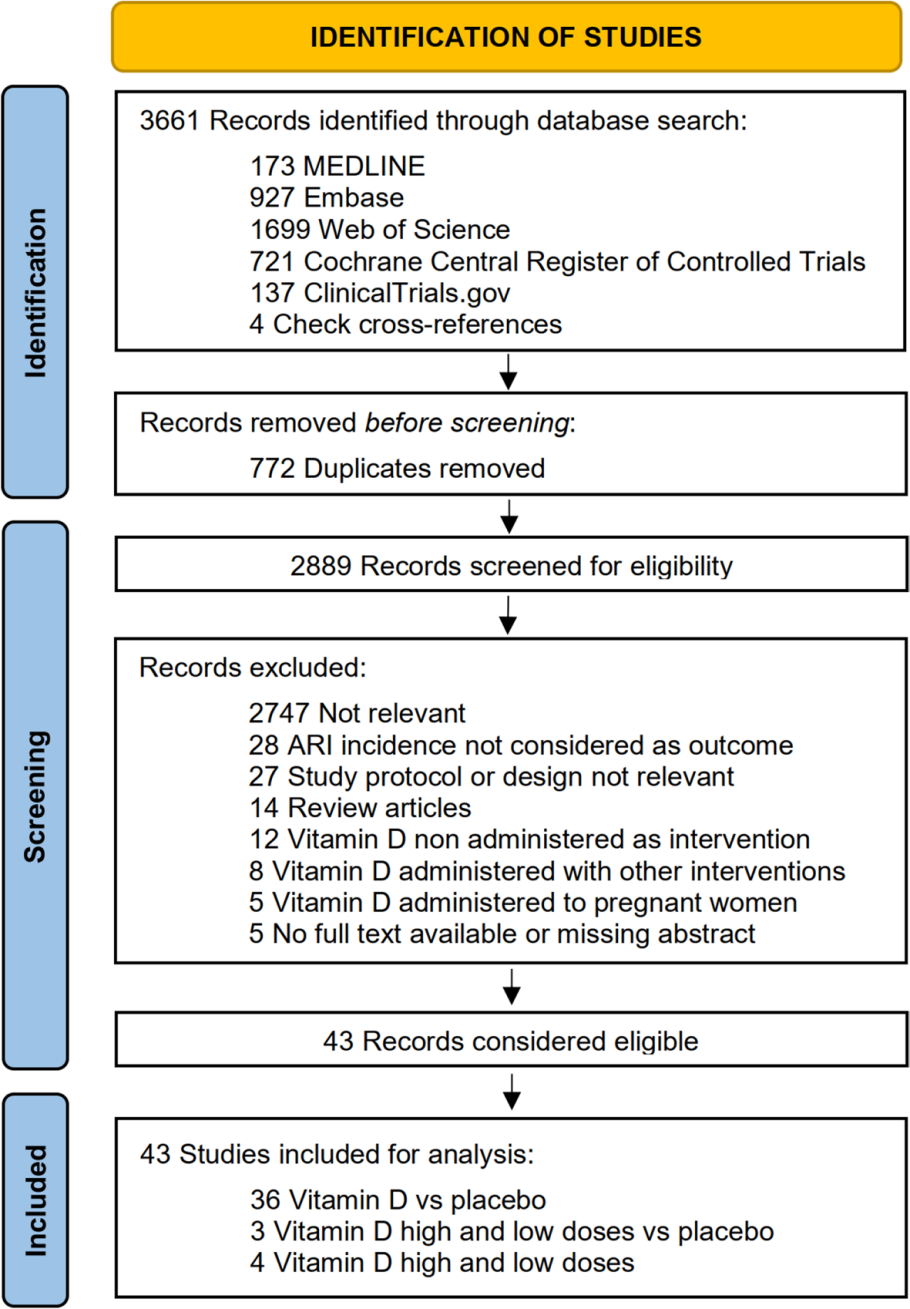
Literature search and selection flow diagram. ARI: acute respiratory infection

As shown in Fig. 1, 43 studies (49320 participants) [28–70] were included in the analysis, of which 36 compared one regimen of vitamin D with placebo [28–41, 44–51, 54, 57–59, 61–70], three compared multiple doses of vitamin D with placebo [42, 43, 60], and four compared two different doses of vitamin D [52, 53, 55, 56]. Table 1 and Supplemental Table 2 shows the characteristics of the included studies. The trials were published from 2009 to 2022, covering five continents with a latitude ranging from 61.04 North to 43.53 South (tropical to temperate zones). Trial durations ranged from 7 weeks to 5 years, involving all four seasons. The participant ages ranged from birth to 95 years, with one trial each studying exclusively for males [29] or females [58]. Thirty-three studies reported the mean baseline 25-hydroxyvitamin D concentrations [28, 29, 33–36, 38–40, 42, 45–50, 52–54, 56, 58–63, 65, 66] [43, 55, 57, 64, 70], with 12 including participants with < 50 nmol/L [35, 36, 46–48, 50, 54] [43, 55, 57, 64, 70]. Twenty-nine studies [28, 29, 31, 35, 37, 38, 41–45, 48, 49, 52–54, 56–58, 60–66, 68–70] were conducted in the general population, while others [30, 32–34, 36, 39] [40, 46, 47, 50, 51, 55] [59, 67] were for disease-specific conditions, such as asthma. Vitamin D was administered daily in 23 studies[28, 29, 31, 33–35, 39, 40, 44, 45, 50, 54, 57–59, 62, 65, 69, 70], weekly in 5 studies [32, 41, 49, 61, 64], monthly in 12 studies [36–38, 43, 46–48, 53, 55, 63, 66, 68], and as a bolus dose in 3 studies [30, 51, 67]. The vitamin D supplementation doses ranged from 200 to 4000 IU/day.

**Table 1:** Main characteristics of the included studies and their participants

Author name, publication year	Number of participants in intervention and control groups^a^	Participants (male %); mean age at baseline in years (age range of inclusion)	Disease specific population	Mean baseline 25(OH)D levels, nmol/L (SD) Percentage of participants with 25(OH)D deficiency (%) with study definition	Oral dose of vitamin D3 in the intervention group (IU) [average daily dose (IU/day)]	Mean 25(OH)D levels after intervention nmol/L (SD)	Country, city Climatic zone	Trial duration Season (from-to)	ARI definition	ARI outcome
Li-Ng et al., 2009 [28]	Intervention = 84 Control = 78	162 (male 21.0%) 57.9 (18–80 years)	No	Intervention 64.3 (25,4) Control 63.0 (25,8) NR	2,000 IU daily vs placebo [2,000 IU/day]	88.5 (23,2)	USA, New York, Temperate	3 Months Winter-Spring	URI: ≥ 2 symptoms and absence of allergy symptoms	Primary
Laaski et al., 2010 [29]	Intervention = 80 Control = 84	164 (male 100%) 19.1 (18–28 years)	No	Intervention 78.7 (14.9) Control 74.4 (20.8) NR	400 IU daily vs placebo [400 IU/day]	71.6 (22.9)	Finland, Huovinrinne, Temperate	6 Months Autumn–Winter	ARI: any acute respiratory tract infection recorded in medical records	Primary
Manaseki-Holland et al., 2010 [30]	Intervention = 224 Control = 229	453 (male 56.7%) 1.1 (1–36 months)	Pneumonia	NM	100,000 IU bolus once vs placebo [1,111 IU/day]	NM	Afghanistan, Kabul, Temperate	3 Months Winter-Spring	LRI: repeat episode of pneumonia (age-specific tachypnoea + no wheeze)	Secondary
Urashima et al., 2010 [31]	Intervention = 217 Control = 213	430 (male 56.3%) 10.2 (6–15 years)	No	NM	1,200 IU daily vs placebo [1,200 IU/day]	NM	Japan, NA, Temperate	4 Months Winter-Spring	URI: RIDT-positive influenza A or B or RIDT- negative influenza like illness	Primary + Secondary
Kumar et al., 2011 [32]	Intervention = 1039 Control = 1040	2079 (male 46.7%) 0.1 (0–48 h)	Low birthweight term infants	NM	1,400 IU weekly vs placebo [200 IU/day]	55.0 (22.5)	India, New Delhi, Temperate	6 Months All year	ARI: episodes leading to hospital admission obtained from medical record	Secondary
Majak et al., 2011 [33]	Intervention = 24 Control = 24	48 (male 50.0%) 10.9 (5–18 years)	Asthma and allergy	Intervention 90.1 (34.7) Control 87.6 (42.2) NR	500 IU daily vs placebo [500 IU/day]	93.9 (32.7)	Poland, Lodz, Temperate	6 Months Autumn-Spring	ARI: self-reported symptoms	Secondary
Bergman et al., 2012 [34]	Intervention = 70 Control = 70	140 (male 27.1%) 53.1 (18–75 years)	Susceptibility to respiratory infections	Intervention 51.5 (NR) Control 46.9 (NR) NR	4,000 IU daily vs placebo [4000 IU/day]	133.4 (NR)	Sweden, Flemingsburg Temperate	12 Months All year	ARI: assessed with questionnaire	Secondary
Camargo et al., 2012 [35]	Intervention = 143 Control = 104	247 (male 52.2%) 10.0 (NR)	No	Intervention 18.0 (3.6) Control 17.1 (3.8) 245/247 (99.2%) (serum 25(OH)D < 50 nmol/L)	300 IU daily vs placebo [300 IU/day]	49.1 (15,1)	Mongolia, Ulaanbaatar, Temperate	7 Weeks Winter-Spring	ARI: parent- reported symptomatic chest infections or colds lasting ≥ 24 h	Secondary
Lehouck et al., 2012 [36]	Intervention = 91; Control = 91	182 (79.7%) 67.9 (> 50 years)	COPD	Intervention 49.9 (30.0) Control 49.9 (27.5) 30/182 (16,5%) (serum 25(OH)D < 25 nmol/L)	100,000 IU bolus monthly vs placebo [3571 IU/day]	128.8 (44.7)	Belgium, Leuven, Temperate	12 Months All year	LRI: self-reported episodes	Secondary
Manaseki- Holland et al., 2012 [37]	Intervention = 1524 Control = 1522	3046 (male 52.2%) 0.5 (1–11 months)	No	NM	100,000 IU bolus every 3 months vs placebo [1111 IU/day]	32.7 (17.1)	Afghanistan, Kabul, Temperate	18 Months All year	LRI: radiologically confirmed pneumonia	Primary
Murdoch et al., 2012 [38]	Intervention = 161 Control = 161	322 (male 25,2%) 48.1 (> 18 years)	No	Intervention 72.4 (22.5) Control 69.9 (22.5) 5/322 (1.6%) (serum 25(OH)D < 25 nmol/L)	200,000 IU bolus every 2 months then 100.000 IU bolus monthly vs placebo [3704 IU/day]	123.6 (27,5)	New Zealand, Christchurch, Temperate	18 Months All year	URI: assessed with questionnaire	Primary
Marchisio et al., 2013 [39]	Intervention = 58 Control = 58	116 (male 55.2%) 2.8 (1–5 years)	History of repeated acute otitis media	Intervention 90.4 (21.2) Control 46.7 (17.7) NR	1,000 IU daily vs placebo [1000 IU/day]	114.6 (19.5)	Italy, Milan, Temperate	6 Months Winter-Spring	URI: doctor diagnosed acute otitis media episodes	Primary
Rees et al., 2013 [40]	Intervention = 401 Control = 360	759 (male 57.7%) 61.2 (45–75 years)	Previous colorectal adenoma (removed)	Intervention 61.9 (20.7) Control 63.2 (22.0) 0 (no definition provided)	1,000 IU daily vs placebo [1000 IU/day]	186.9 (455.1)	USA, NA Temperate	13 Months All year	URI: assessed from patient’s diary	Secondary
Goodall et al., 2014 [41]	Intervention = 300 Control = 300	600 (male 36.3%) 19.6 (> 17 years)	No	NM	10,000 IU weekly vs placebo [1429 IU/day]	NM	Canada, Hamilton, Temperate	8 Weeks Autumn	URI: self-reported symptomatic cold	Primary
Grant et al., 2014 [42]	Intervention Group 1 = 87 Group 2 = 86 Control = 87	249 (male 48.6%) unborn at baseline	No	NR	Group 1: 400 IU Group 2: 800 IU daily vs placebo [G1: 400 IU/day] [G2: 800 IU/day]	Group 1 85.2 (34.7) Group 2 101.1 (46.8)	New Zealand, Auckland, Temperate	6 Months All year	ARI: doctor diagnosed ARI during primary care visits	Secondary
Tran et al., 2014 [43]	Intervention Group 1 = 215 Group 2 = 215 Control = 214	644 (male 53.3%) 71.7 (60–84 years)	No	Group 1: 41.5 (12.8) Group 2: 41.5 (14.1) Control 41.9 (13.2) 61/620 (9.8%) (serum 25(OH)D < 25 nmol/L)	Group 1:30,000 IU Group 2:60,000 IU bolus monthly vs placebo [G1: 1000 IU/day] [G2: 2000 IU/day]	Group 1 64.0 (16.8) Group 2 77.9 (19.9)	Australia, Queensland, New South Wales, Victoria, Tasmania Temperate	12 Months All year	URI: assessed with questionnaire and medical records	Secondary
Urashima et al., 2014 [44]	Intervention = 148 Control = 99	247 (male 65.6%) 16.5 (15–18 years)	No	NM	2,000 IU daily vs placebo [2000 IU/day]	NM	Japan, Tokyo, Temperate	2 Months Winter	URI: RIDT-positive influenza A or RIDT- negative influenza like illness	Primary
Dubnov-Raz et al., 2015 [45]	Intervention = 28 Control = 27	54 (male 63%) 15·2 (12–21 years)	No	Intervention 60.7 (12.2) Control 60.9 (11.7) 11/54 (20.4%) (serum 25(OH)D < 50 nmol/L)	2,000 IU daily vs placebo [2000 IU/day]	74.6 (16.2)	Israel, Petah-Tikva, Temperate	3 Months Winter	URI: assessed with symptom score	Primary
Martineau et al., 2015, ViDiAs Trial, [46]	Intervention = 125 Control = 125	250 (male 43.6%) 47.9 (16–78 years)	Asthma	Intervention 49.8 (25.2) Control 49.4 (24.2) 36/250 (14·4%) (serum 25(OH)D < 25 nmol/L)	120,000 IU bolus once every 2 months vs placebo [2000 IU/day]	69.4 (21.0)	England, London, Temperate	12 Months All year	URI: assessed from daily symptom scores diary	Coprimary
Martineau et al., 2015, ViDiCO Trial, [47]	Intervention = 122 Control = 118	240 (male 60%) 64.7 (> 40 years)	COPD	Intervention 45.4 (27.9) Control 46.7 (23.3) 50/240 (20.8%) (serum 25(OH)D < 25 nmol/L)	120,000 IU bolus every 2 months vs placebo [2000 IU/day]	67.4 (27.5)	England, London, Temperate	12 Months All year	URI: assessed from patient’s diary	Coprimary
Martineau et al., 2015, ViDiFlu Trial [48]	Intervention = 137 Control = 103	240 (male 34.2%) 67.1 (21.4–94.0 years)	No	Intervention 42.4 (23.4) Control 43.6 (22.6) 60/240 (25%) (serum 25(OH)D < 25 nmol/L)	Resident: 96,000 IU bolus every 2 months + 400 IU daily; Carers: 120,000 IU bolus every 2 months vs controls [2000 IU/day]^d^	82.8 (4.4)	England, London, Temperate	12 Months All year	URI and LRI: both assessed from daily symptom diary	Primary + Secondary
Simpson et al., 2015 [49]	Intervention = 18 Control = 16	34 (male 41.2%) 32.2 (18–52 years)	No	Intervention 60.5 (13.9) Control 76.4 (27.3) 8/34 (23.5%) (serum 25(OH)D < 50 nmol/L) 4/34 (11.8%) (serum 25(OH)D < 40 nmol/L)	20,000 IU weekly vs placebo [2857 IU/day]	100.7 (23.9)	Australia, Hobart, Temperate	17 Weeks Autumn-Spring	ARI: assessed with symptom score	Primary
Denlinger et al., 2016 [50]	Intervention = 201 Control = 207	408 (male 31.9%) 39.2 (18–85 years)	Asthma	Intervention 45.1 (12.5) Control 49.2 (12.5) 111/203 (54.7%) (serum 25(OH)D < 50 nmol/L)	Once 100,000 IU bolus, then 4,000 IU daily vs placebo [4000 IU/day]	104.6 (4.5)	USA, NA, Temperate	7 Months Winter-Summer	URI: assessed with symptom score	Secondary
Gupta et al., 2016 [51]	Intervention = 162 Control = 162	324 (male 69.8%) 1.4 (0.5–5 years)	Severe pneumonia	Intervention 35.9 (19.5) Control 38.2 (19.1) 126/324 (38.9%) (serum 25(OH)D < 30 nmol/L)	One 100,000 IU bolus vs placebo [556 IU/day]	NA	India, New Delhi, Temperate	6 Months All year	ARI: physician confirmed recurrent pneumonia	Coprimary
Aglipay et al., 2017 [52]	Intervention = 349 Control = 354	703 (male 57.5%) 2.7 (1–5 years)	No	Intervention 89.6 (30.7) Control 92.1 (29.2) NM	2,000 IU daily vs 400 IU daily [2000 IU/day]	121.6 (4.5)	Canada, Toronto, Temperate	4–8 Months Autumn-Spring	URI: laboratory confirmed	Primary
Ginde et al., 2017 [53]	Intervention = 55 Control = 52	107 (male 42.1%) 80.7 (60–95 years)	No	Intervention 57.4 (21.0) Control 57.4 (24.7) 37/107 (34.6%) (serum 25(OH)D < 50 nmol/L)	100,000 IU bolus monthly vs 12,000 IU bolus monthly (or Placebo + 400–1,000 IU per day equivalent) [3333 IU/day]	81.4	USA, Aurora, Temperate	12 Months All year	ARI: medical record diagnosis by nurse or physician assessment and/or new prescribed treatment	Primary
Hibbs et al., 2018 [54]	Intervention = 153 Control = 147	300 (male 55.3%)^b^ unborn at baseline	No	Intervention 47.7 (NR) Control 52.4 (NR)^c^ 0% (serum 25(OH)D < 25 nmol/L)	400 IU daily vs placebo [400 IU/day]	NA	USA, Cleveland, Temperate	12 Months All year	ARI: self-reported URI or LRI, assessed by questionnaire	Secondary
Lee et al., 2018 [55]	Intervention = 31 Control = 31	62 (male 48.4%) 9.9 (3–20 years)	Sickle cell disease	Intervention 37.4 (17.5) Control 33.9 (15.5) 48/62 (77.4%) (serum 25(OH)D < 50 nmol/L)	100,000 IU bolus monthly vs 12,000 IU bolus monthly [3333 IU/day]	90.1 (NM)	USA, New York, Temperate	24 Months All year	Self-reported respiratory events, including ARI	Primary
Rosendhal et al., 2018 [56]	Intervention = 492 Control = 495	987 (male 50.2%) unborn at baseline	No	Intervention 81.3 (24.0) Control 81.7 (27.8) 41/955 (4.3%) (serum 25(OH)D < 50 nmol/L)	1,200 IU daily vs 400 IU daily [1200 IU/day]	117.7(26.1)	Finland, Helsinki, Temperate	24 Months All year	Parent-reported infections, including ARI	Coprimary
Shimizu et al., 2018 [57]	Intervention = 126 Control = 126	252 (male 32.5%) 53.1 (45–74 years)	No	Intervention 49.2 (13.8) Control 48.9 (13.0) 121/215 (56.3%) (serum 25(OH)D < 50 nmol/L)	400 IU daily vs placebo [400 IU/day]	114.6 (32.7)	Japan, Tokyo, Yokohama, Temperate	4 Months Winter-Summer	ARI: self-reported assessed by questionnaire	Primary
Aloia et al., 2019 [58]	Intervention = 130 Control = 130	260 (male 0%) 68.2 (65.4–72.5 years)	No	Intervention 53.7 (16.2) Control 55.4 (17.2) NM	2,000 IU daily vs placebo [2000 IU/day]	117.3 (28.0)	USA, New York, Temperate	3 Months All year	ARI: self-reported common cold or influenza	Secondary
Arihiro et al., 2019 [59]	Intervention = 119 Control = 118	237 (male 61.6%) 44.7 (18–80 years)	Ulcerative Colitis or Crohn’s Disease	Intervention 57.4 (18.2) Control 59.7 (25.5) 77/223 (34.5%) (serum 25(OH)D < 50 nmol/L)	500 IU daily vs placebo [500 IU/day]	80.4 (NR)	Japan, Tokyo, Temperate	6 Months Winter-Spring	ARI: laboratory confirmed influenza, URI: diagnosed by clinician	Primary + Secondary
Hauger et al., 2019 [60]	Intervention Group 1 = 44 Group 2 = 43 Control = 43	130 (male 46.9%) 6.7 (4–8 years)	No	Group 1: 56.9 (12.7) Group 2: 58.1 (13.5) Control 55.2 (10.8) NM	Group 1: 400 IU Group 2: 800 IU daily vs placebo [G1: 400 IU/day] [G2: 800 IU/day]	Group 1 61.8 (10.6) Group 2 75.8 (11.5)	Denmark, Copenhagen, Temperate	5 Months Autumn-Spring	ARI: self-reported	Secondary
Loeb et al., 2019 [61]	Intervention = 650 Control = 650	1300 (male 47.8%) 8.5 (3–17 years)	No	Intervention 65.7 (16.7) Control 65.2 (16.9) 6/1300 (0.5%) (serum 25(OH)D < 25 nmol/L)	14,000 IU weekly vs placebo [2000 IU/day]	91.8 (23.6)	Vietnam, Hanoi, Tropical	8 Months All year	ARI: RT-PCR confirmed influenza A or B	Primary
Bischop-Ferrari et al., 2020 [62]	Intervention = 1076 Control = 1081	2157 (male 38.3%) 74.9 (70–95 years)	No	Intervention 55.9 (21.0) Control 55.9 (21.2) 241/2140 (11.3%) (serum 25(OH)D < 30 nmol/L)	2,000 IU daily vs placebo [2000 IU/day]^e^	93.9 (NR)	Switzerland, France, Austria, Germany, Portugal, NA, Temperate	3 Years All year	ARI: self-reported and verified by independent physician	Coprimary
Camargo et al., 2020 [63]	Intervention = 2558 Control = 2552	5056 (male 58%) 66.4 (50–84)	No	Intervention 63.7 (23.6) Control 63.0 (23.5) 89/5056 (2.0%) (serum 25(OH)D < 25 nmol/L)	200,000 IU bolus followed by a monthly 100, 000 IU vs placebo, [3300 IU/day]	135 (NA)	New Zealand, NA, Temperate	3 Years All year	Self-reported: cold, runny nose, sore throat, flu-like illness, or chest infection	Secondary
Ganmaa et al., 2020 [64]	Intervention = 4418 Control = 4433	8851 (male 51%) 9.4 (6–13)	No	Intervention 29.7 (10.5) Control 29.7 (10.5) 2813/8846 (31.8%) (serum 25(OH)D < 25 nmol/L)	14,000 IU weekly vs placebo, [2000 IU/day]	77.4 (22.7)	Mongolia, Ulaanbaatar, Temperate	3 Years All year	Self-reported	Secondary
Mandlik et al., 2020 [65]	Intervention = 135 Control = 150	244 (male 53%) 8.0 (6–12)	No	Intervention 60.2 (11.9) Control 57.7 (10.0) NA	1,000 IU daily vs placebo, [1000 IU/day]	80.0 (23.3)	India, Pune, Tropical	6 Months Summer–Winter	Self-reported	Primary
Rake et al., 2020 [66]	Intervention = 395 Control = 392	787 (male NA) NA (65–84)	No	NA 127/781 (16.3%) (serum 25(OH)D < 25 nmol/L)	100,000 IU bolus monthly vs placebo, [3300 IU/day]	109.2 (NR)	England, NA, Temperate	2 Years All year	Reported by general practitioner	Secondary
Jadhav et al., 2021 [67]	Intervention = 155 Control = 155	298 (male 61.3%) 3.0 (1–5)	Recurrent ARI	NM	120,000 IU bolus vs placebo [667 IU/day]	NM	India, Karad, Tropical	6 Months All year	ARI: self-reported	Primary
Pham et al., 2021 [68]	Intervention = 8000 Control = 8000	15,373 (male 54.3%) NA 60–84)	No	NM	60,000 IU bolus monthly vs placebo, [2000 IU/day]	114.8 (30.0)	Australia, NA, Temperate	5 Years All year	Self-reported: cold, runny nose, sore throat, the flu	Secondary
Huang et al., 2022 [69]	Intervention = 135 Control = 113	248 (male 69.3%) 3.9 (2–5)	No	NM	2000 IU daily vs placebo [2000 IU/day]	NM	Taiwan, North and South, Temperate + Tropical	6 Months All year	ARI: lab-confirmed influenza illness	Primary
Villasis-Keever et al., 2022 [70]	Intervention = 161 Control = 160	321 (male 30%) 37.5 (NR)	No	Intervention 18.3 (NR) Control 17.1 (NR)^c^ 215/321 (67.0%) (serum 25(OH)D < 50 nmol/L)	4000 IU daily vs placebo [4000 IU/day]	67.4 (NR)	Mexico, Mexico City, Tropical	45 Days Summer–Winter	ARI: positive laboratory result for SARS-CoV-2 infection	Primary

*25(OH)D* 25-hydroxyvitamin D, *ARI* Acute respiratory infection, *NA* Not applicable, *NM* Not measured, *NR* Not reported, *RIDT* Rapid influenza diagnostic test, *URI* Upper respiratory infections

^a^Based on the intention-to-treat original study number

^b^Sex was missing for one participant

^c^Reported the median with interquartile range, not the mean and standard deviation

^d^Controls: carers assumed placebo; residents assumed placebo + 400 IU of 25(OH)D

^e^Trial design: Vitamin D (2 × 2 × 2 factorial with omega-3 fatty acid supplementation and strength training exercise)

Supplemental Table 3 demonstrates that all trials were considered at low risk of bias for all five domains assessed, except for three trials [29, 45, 67] with an unclear risk of bias due to a high percentage of outcome data lost during follow-up.

### Main analysis

**Fig. 2:**
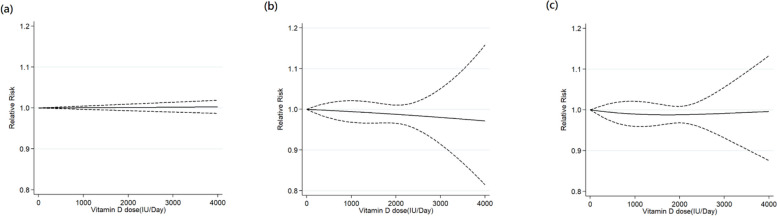
Model comparison of main dose–response meta-analysis. The solid black line indicates the linear model (**a**), the quadratic model (**b**), and the restricted cubic spline model (**c**). Dashed black lines are 95% point-wise confidence intervals estimated by the respective 1-stage random-effects model. The Akaike Information Criterion values for each model are (**a**) -3.21, (**b**) 36.56 and (**c**) 15.81

**Fig. 3:**
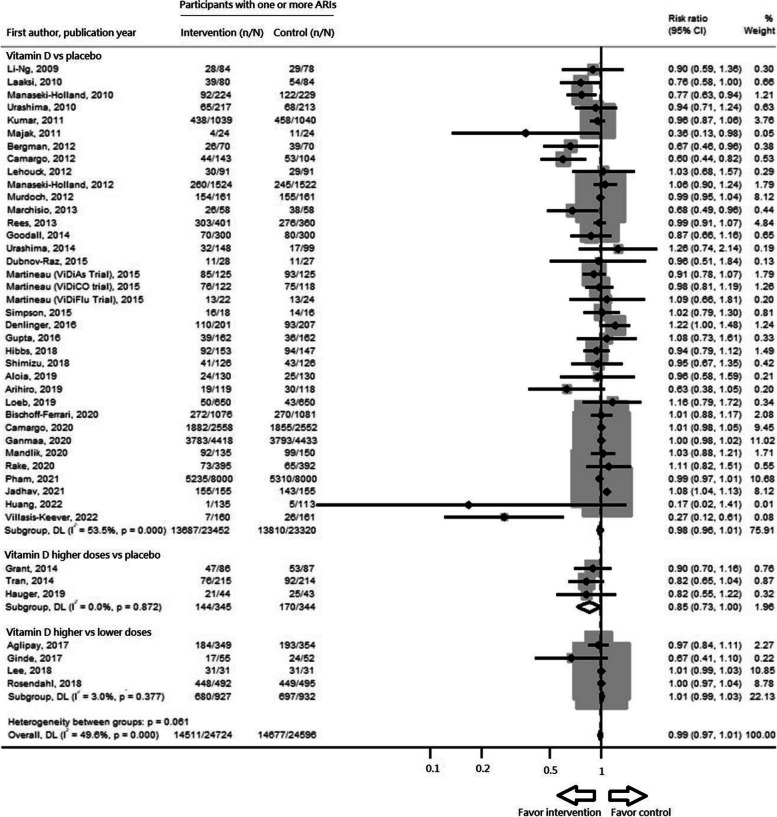
Main pairwise meta-analysis including all eligible studies based on random-effects model. Forest plot of the summary risk ratios comparing proportions of participants with one or more ARIs between intervention and control groups. In the comparison of vitamin D higher doses vs placebo, there were two or more levels of vitamin D doses in each included study; only the group with highest vitamin D dose and the placebo in each study were selected for pooling. In the comparison of vitamin D higher vs lower doses, there were no placebo control group in included studies; the two groups with different vitamin D doses in each study were selected for pooling. CI: confidence interval; DL: DerSimonian and Laird random effects model; n: number of participants with one or more ARI; N:total number of participants in the study group

The dose–response meta-analysis tested three models: linear, quadratic, and restricted cubic spline (Fig. 2). Compared to the quadratic model, the restricted cubic spline model exhibited lower Akaike Information Criterion (AIC) values, suggesting a J-shaped association between the dose of vitamin D supplementation and its preventive effects. Nonetheless, no significant preventive effects were noted at pre-specified vitamin D supplementation doses (Table 2, Fig. 2). The pairwise meta-analysis indicated there were no significant preventive effects of vitamin D supplementation against ARIs (RR: 0.99, 95% confidence interval [CI]: 0.97–1.01, *I*^*2*^ = 49.6%, *p* for heterogeneity (*p*_*het*_) < 0.001) (Table 2, Fig. 3). Even when stratified by the comparators, no significant preventive effects were observed in the three comparison groups, including vitamin D vs placebo, higher doses vs placebo, or higher vs lower doses.

**Table 2:** Results of pairwise and dose–response meta-analysis

Group	Study number; Patient number	Dose–response meta-analysis, RR (95% CI)			Pairwise Meta-analysis, RR (95% CI), NNT
		400 IU/d	800 IU/d	1200 IU/d	
**Main analysis**					
All [28–70]	43; 49,320	0.99 (0.98–1.01)	0.99 (0.96–1.02)	0.99 (0.96–1.02)	0.99 (0.97–1.01)
**Subgroup analysis**					
* Age group (years)*					
< 7 [30, 32, 37, 39, 42, 51, 52, 54, 56, 60, 67, 69]	12; 8826	0.95 (0.87–1.03)	0.93 (0.83–1.04)	0.93 (0.84–1.03)	0.97 (0.91–1.13)
7–17 [31, 33, 35, 44, 45, 55, 61, 64, 65]	9; 11,525	NA	NA	NA	1.00 (0.96–1.04)
18–65 [28, 29, 34, 38, 40, 41, 46, 47, 49, 50, 57, 59, 70]	13; 3891	0.97 (0.91–1.03)	0.95 (0.86–1.05)	0.93 (0.84–1.04)	0.94 (0.86–1.02)
> 65 [36, 43, 48, 53, 58, 62, 63, 66, 68]	9; 25,078	0.99 (0.97–1.01)	0.98 (0.95–1.01)	0.98 (0.94–1.01)	0.99 (0.98–1.01)
* Gender proportion (%)*					
Male > 60 [29, 33, 36, 41, 44, 45, 51, 67]	8; 1930	0.92 (0.74–1.13)	0.93 (0.77–1.13)	0.95 (0.80–1.12)	0.96 (0.82–1.13)
Male ≤ 60 [28, 30–32, 34, 35, 37–40, 42, 43, 46–50, 52–66, 68–70]	35; 47,390	0.99 (0.99–1.00)	0.99 (0.98–1.00)	0.99 (0.98–1.00)	0.99 (0.96–1.01)
* Comorbidity*					
General [28, 29, 31, 35, 37, 38, 41–45, 48, 49, 52–54, 56–58, 60–66, 68–70]	29; 43,560	1.00 (0.99–1.01)	0.99 (0.98–1.01)	0.99 (0.98–1.01)	0.99 (0.97–1.01)
Disease-specific [30, 32–34, 36, 39, 40, 46, 47, 50, 51, 55, 59, 67]	14; 5610	0.94 (0.88–1.01)	0.92 (0.83–1.01)	0.91 (0.81–1.02)	0.97 (0.91–1.03)
* Baseline 25-hydroxyvitamin D levels (nmol/L)*					
< 50 [35, 36, 43, 46–48, 50, 54, 55, 57, 64, 70]	12; 11,588	0.99 (0.98–1.01)	0.99 (0.96–1.02)	0.99 (0.96–1.02)	0.98 (0.94–1.03)
> 50 [28, 29, 33, 34, 38–40, 42, 45, 49, 52, 53, 56, 58–63, 65, 66]	21; 13,995	0.99 (0.98–1.01)	0.99 (0.96–1.02)	0.99 (0.96–1.02)	0.98 (0.95–1.02)
* Dosing frequency*					
Bolus [30, 51, 67]	3; 1087	NA	NA	NA	0.96 (0.75–1.24)
Daily [28, 29, 31, 33–35, 39, 40, 42, 44, 45, 50, 52, 54, 56–60, 62, 65, 69, 70]	23; 8788	0.94 (0.87–1.02)	0.92 (0.82–1.02)	0.92 (0.84–1.02)	0.92 (0.85–0.99), 36
Weekly [32, 41, 49, 61, 64]	5; 12,864	NA	NA	NA	1.00 (0.98–1.02)
Monthly [36–38, 43, 46–48, 53, 55, 63, 66, 68]	12; 26,581	0.99 (0.98–1.00)	0.99 (0.97–1.00)	0.98 (0.96–1.00)	1.00 (0.99–1.01)
* Trial duration (months)*					
< 4 [28, 30, 35, 41, 44, 45, 57, 58, 69, 70]	10; 2845	0.86 (0.69–1.07)	0.80 (0.60–1.07)	0.81 (0.63–1.04)	0.81 (0.67–0.97), 16
4–12 [29, 31–33, 39, 42, 49–52, 59–61, 65, 67]	15; 6698	0.92 (0.85–1.01)	0.91 (0.81–1.02)	0.93 (0.83–1.03)	0.97 (0.89–1.05)
> 12 [34, 36–38, 40, 43, 46–48, 53–56, 62–64, 66, 68]	18; 39,777	NA	NA	NA	1.00 (0.99–1.01)
* Climatic zone*					
Tropical or Subtropical [61, 65, 67, 69, 70]	5; 2464	1.13 (1.00–1.29)	1.06 (0.97–1.16)	0.97 (0.82–1.15)	0.97 (0.77–1.21)
Temperate [28–60, 62–64, 66, 68]	38; 46,856	0.99 (0.99–1.00)	0.99 (0.98–1.00)	0.99 (0.97–1.00)	0.99 (0.97–1.01)
* Summer*					
Summer-inclusive [32, 34, 36–38, 40, 42, 43, 46–49, 51, 52, 54–59, 62–70]	29; 44,896	1.01 (0.99–1.02)	1.01 (0.98–1.04)	1.01 (0.98–1.03)	1.00 (0.98–1.02)
Summer-sparing [28–31, 33, 35, 39, 41, 44, 45, 50, 53, 60, 61]	14; 4424	0.83 (0.75–0.92)	0.77 (0.67–0.88)	0.79 (0.69–0.90)	0.85 (0.74–0.98), 26
* Winter*					
Winter-dominant [28–31, 35, 39, 44, 45, 60]	9; 1961	0.72 (0.62–0.82)	0.70 (0.61–0.81)	0.80 (0.71–0.90)	0.79 (0.71–0.89), 10
Winter-non-dominant [32–34, 36–38, 40–43, 46–59, 61–70]	34; 47,359	1.00 (0.99–1.02)	1.01 (0.98–1.03)	1.00 (0.98–1.03)	1.00 (0.98–1.02)
**Sensitivity analysis**					
* Type of ARIs*					
Mixed upper and lower respiratory tract infections [29, 32–35, 42, 48, 49, 53–56, 60, 62–67]	19; 21,974	1.00 (0.98–1.03)	1.01 (0.97–1.05)	1.01 (0.97–1.06)	1.00 (0.97–1.03)
Upper respiratory tract infections [28, 38–41, 43, 45–47, 50, 52, 54, 57–59, 61, 68]	17; 22,395	0.99 (0.94–1.00)	0.99 (0.91–1.00)	0.99 (0.92–1.00)	0.98 (0.96–1.01)
Lower respiratory tract infections [30, 36, 37, 51, 54]	5; 4305	0.98 (0.80–1.12)	0.95 (0.82–1.09)	0.95 (0.79–1.13)	0.95 (0.83–1.08)
Influenza [31, 44, 52, 53, 55, 58, 59, 61, 69]	9; 3594	0.98 (0.88–1.08)	0.96 (0.80–1.13)	0.96 (0.80–1.15)	0.98 (0.89–1.07)

The dose–response meta-analysis could not achieve convergence on age group: 7–17 years, dosing frequency: weekly, and trial duration: > 12 months. The sample size was too small for dosing frequency: bolus to perform dose–response analysis. NNT was calculated for those meta-analyses showing significant preventive results

*ARI* Acute respiratory infection, *NA* Not available, *NNT* Number needed to treat

### Subgroup and sensitivity analysis

Subgroup analyses (Table 2 and Supplemental Figs. 1–10) were performed to investigate whether vitamin D supplementation may be more effective in specific subgroups. The dose–response meta-analysis identified that the optimal vitamin D supplementation doses ranged between 400–1200 IU/d for both summer-sparing and winter-dominant subgroups (Table 2 and Supplemental Figs. 8 and 9). The pairwise meta-analysis further revealed significant preventive effects of vitamin D supplementation in subgroups of daily dosing (RR: 0.92, 95% CI: 0.85–0.99,* I*^*2*^ = 55.7%, *p*_*het*_ = 0.001, NNT = 36), trials duration < 4 months (RR: 0.81, 95% CI: 0.67–0.97, *I*^*2*^ = 48.8%, *p*_*het*_ = 0.04, NNT = 16), summer-sparing seasons (RR: 0.85, 95% CI: 0.74–0.98,* I*^*2*^ = 55.8%, *p*_*het*_ = 0.006, NNT = 26), and winter-dominant seasons (RR: 0.79, 95% CI: 0.71–0.89, *I*^*2*^ = 9.7%, *p*_*het*_ = 0.35, NNT = 10). Finally, the number of studies defining ARIs as lower respiratory tract infections or influenza was substantially lower than those defining ARIs as either combined upper and lower respiratory tract infections or solely upper respiratory tract infections. The sensitivity analysis indicated no significant preventive effects of vitamin D supplementation for any specific ARIs. Also, when pooling studies according to different dosing frequencies, the sensitivity analyses indicated that the synthesized results for daily or weekly vitamin D supplementation remained consistent with those of the main analysis (Tables 3 and 4). In contrast, for bolus or monthly vitamin D supplementation, no obvious preventative effects of vitamin D supplementation were observed (Table 5).

**Table 3:** Sensitivity analysis for daily supplementation of vitamin D

Group	Study number; Patient number	Dose–response meta-analysis, RR (95% CI)			Pairwise Meta-analysis, RR (95% CI)
		400 IU/d	800 IU/d	1200 IU/d	
**Sensitivity analysis**					
Daily administration [28, 29, 31, 33–35, 39, 40, 42, 44, 45, 50, 52, 54, 56–60, 62, 65, 69, 70]	23; 8788	0.94 (0.87–1.02)	0.92 (0.82–1.02)	0.92 (0.84–1.02)	0.92 (0.85–0.99)
**Subgroups**					
* Age group (years)*					
< 7 [39, 42, 52, 54, 56, 60, 69]	7; 2614	0.88 (0.79–0.99)	0.87 (0.77–0.98)	0.88 (0.78–0.98)	0.92 (0.84–1.01)
7–17 [31, 33, 35, 44, 45, 65]	6; 1312	0.70 (0.50–1.00)	0.81 (0.64–1.03)	0.94 (0.77–1.14)	0.87 (0.67–1.12)
18–65 [28, 29, 34, 40, 50, 57, 59, 70]	8; 2445	0.93 (0.76–1.12)	0.89 (0.72–1.11)	0.88 (0.72–1.06)	0.84 (0.70–1.02)
> 65 [58, 62]	2; 2417	NA	NA	NA	0.91 (0.85–0.98)
* Gender proportion (%)*					
Male > 60[29, 33, 44, 45]	4; 514	NA	NA	NA	0.84 (0.58–1.21)
Male ≤ 60 [28, 31, 34, 35, 39, 40, 42, 50, 52, 54, 56–60, 62, 65, 69, 70]	19; 8274	0.97 (0.91–1.02)	0.95 (0.88–1.03)	0.95 (0.88–1.02)	0.92 (0.86–0.99)
* Comorbidity*					
General [28, 29, 31, 35, 42, 44, 45, 52, 54, 56–58, 60, 62, 65, 69, 70]	17; 7078	0.95 (0.86–1.05)	0.93 (0.81–1.07)	0.94 (0.84–1.06)	0.92 (0.85–0.99)
Disease-specific [33, 34, 39, 40, 50, 59]	6; 1710	0.70 (0.49–1.01)	0.73 (0.53–1.00)	0.75 (0.56–1.00)	0.83 (0.66–1.04)
* Baseline 25-hydroxyvitamin D levels (nmol/L)*					
< 50 [35, 50, 54, 57, 70]	5; 1528	0.84 (0.64–1.10)	0.81 (0.60–1.10)	0.79 (0.53–1.20)	0.81 (0.59–1.11)
> 50 [28, 29, 34, 39, 40, 42, 45, 52, 56, 58–60, 62, 65, 66]	15; 6335	0.95 (0.88–1.03)	0.94 (0.84–1.04)	0.94 (0.85–1.03)	0.93 (0.87–0.99)
* Trial duration (months)*					
< 4 [28, 35, 44, 45, 57, 58, 69, 70]	8; 1792	0.90 (0.52–1.57)	0.87 (0.41–1.82)	0.87 (0.75–1.69)	0.79 (0.59–1.05)
4–12 [29, 31, 33, 39, 42, 50, 52, 59, 60, 65]	10; 2651	0.86 (0.76–0.97)	0.84 (0.72–0.97)	0.87 (0.76–1.00)	0.89 (0.79–1.00)
> 12 [34, 40, 54, 56, 62]	5; 4345	NA	NA	NA	0.99 (0.94–1.04)
* Climatic zone*					
Tropical or Subtropical [65, 69, 70]	3; 854	NA	NA	NA	0.45 (0.14–1.48)
Temperate [28, 29, 31, 33–35, 39, 40, 42, 44, 45, 50, 52, 54, 56–60, 62]	20; 7934	0.93 (0.86–1.00)	0.90 (0.81–0.99)	0.91 (0.82–1.00)	0.92 (0.86–0.98)
* Summer*					
Summer-inclusive [34, 40, 42, 52, 54, 56–59, 62, 65, 69, 70]	13; 6824	1.00 (0.96–1.05)	1.00 (0.94–1.06)	0.98 (0.92–1.05)	0.96 (0.90–1.02)
Summer-sparing [28, 29, 31, 33, 35, 39, 44, 45, 50, 60]	10; 1964	0.75 (0.65–0.87)	0.72 (0.60–0.85)	0.78 (0.67–0.91)	0.83 (0.69–0.99)
* Winter*					
Winter-dominant [28, 29, 31, 35, 39, 44, 45, 60] [28, 29, 31, 35, 39, 44, 45, 60]	8; 1658	0.69 (0.58–0.82)	0.70 (0.59–0.82)	0.80 (0.69–0.93)	0.78 (0.68–0.92)
Winter-non-dominant [33, 34, 40, 42, 50, 52, 54, 56–59, 62, 65, 69, 70]	15; 7130	1.00 (0.95–1.04)	0.99 (0.93–1.06)	0.98 (0.92–1.05)	0.96 (0.89–1.02)
**Sensitivity analysis**					
* Type of ARIs*					
Mixed upper and lower respiratory tract infections [29, 33–35, 42, 54, 56, 60, 62, 65]	10; 4588	0.87 (0.74–1.01)	0.87 (0.74–1.01)	0.90 (0.79–1.02)	0.88 (0.80–0.97)
Upper respiratory tract infections [28, 39, 40, 45, 50, 52, 54, 57–59]	10; 3254	0.91 (0.83–1.00)	0.87 (0.75–1.00)	0.87 (0.74–1.01)	0.97 (0.89–1.05)
Lower respiratory tract infections [54]	1; 300	NA	NA	NA	0.94 (0.79–1.12)
Influenza [31, 44, 52, 58, 59, 69]	6; 2125	0.90 (0.71–1.14)	0.87 (0.64–1.18)	0.89 (0.69–1.16)	0.94 (0.80–1.10)

*ARI* Acute respiratory infection, *NA* Not available

**Table 4:** Sensitivity analysis for daily or weekly supplementation of vitamin D

Group	Study number; Patient number	Dose–response meta-analysis, RR (95% CI)			Pairwise Meta-analysis, RR (95% CI)
		400 IU/d	800 IU/d	1200 IU/d	
**Sensitivity analysis**					
Daily or weekly administration [28, 29, 31–35, 39–42, 44, 45, 49, 50, 52, 54, 56–62, 64, 65, 69, 70]	28; 21,652	0.95 (0.90–1.01)	0.93 (0.86–1.01)	0.94 (0.87–1.01)	0.95 (0.91–0.99)
**Subgroups**					
* Age group (years)*					
< 7 [32, 39, 42, 52, 54, 56, 60, 69]	8; 4693	0.90 (0.82–0.98)	0.88 (0.79–0.98)	0.89 (0.81–0.98)	0.94 (0.88–1.01)
7–17 [31, 33, 35, 44, 45, 61, 64, 65]	8; 11,463	0.68 (0.00–1.3e + 24)	0.64 (0.00–1.5e + 27)	0.73 (0.00–2.2e + 19)	0.95 (0.82–1.09)
18–65 [28, 29, 34, 40, 41, 49, 50, 57, 59, 70]	10; 3079	0.94 (0.84–1.05)	0.90 (0.76–1.06)	0.88 (0.74–1.04)	0.88 (0.76–1.02)
> 65[58, 62]	2; 2417	NA	NA	NA	1.01 (0.88–1.16)
* Gender proportion (%)*					
Male > 60 [29, 33, 41, 44, 45]	5; 1114	0.63 (0.39–1.01)	0.72 (0.52–1.01)	0.83 (0.66–1.05)	0.85 (0.67–1.07)
Male ≤ 60 [28, 31, 32, 34, 35, 39, 40, 42, 49, 50, 52, 54, 56–62, 64, 65, 69, 70]	23; 20,538	0.97 (0.92–1.03)	0.96 (0.89–1.04)	0.96 (0.90–1.03)	0.96 (0.92–1.00)
* Comorbidity*					
General [28, 29, 31, 35, 41, 42, 44, 45, 49, 52, 54, 56–58, 60–62, 64, 65, 69, 70]	21; 17,863	0.92 (0.00–3.2e + 26)	0.89 (0.00–1.9e + 37)	0.90 (0.00–1.4e + 33)	0.92 (0.85–0.99)
Disease-specific [32–34, 39, 40, 50, 59]	7; 3789	0.81 (0.64–1.04)	0.82 (0.67–1.02)	0.84 (0.68–1.03)	0.90 (0.78–1.04)
* Baseline 25-hydroxyvitamin D levels (nmol/L)*					
< 50 [35, 50, 54, 57, 64, 70]	6; 10,379	0.85 (0.65–1.12)	0.86 (0.68–1.09)	0.87 (0.67–1.11)	0.90 (0.75–1.07)
> 50 [28, 29, 33, 34, 39, 40, 42, 45, 49, 52, 56, 58–62, 65]	17; 7669	0.97 (0.91–1.03)	0.95 (0.87–1.04)	0.95 (0.88–1.04)	0.94 (0.89–1.00)
* Trial duration (months)*					
< 4 [28, 35, 41, 44, 45, 57, 58, 69, 70]	9; 2392	0.88 (0.62–1.26)	0.84 (0.52–1.35)	0.84 (0.55–1.29)	0.81 (0.64–1.02)
4–12 [29, 31–33, 39, 42, 49, 50, 52, 59–61, 65]	13; 6064	0.89 (0.82–0.98)	0.87 (0.77–0.98)	0.89 (0.80–0.99)	0.93 (0.85–1.01)
> 12 [34, 40, 54, 56, 62, 64]	6; 13,196	1.00 (0.92–1.09)	1.00 (0.92–1.10)	1.00 (0.94–1.07)	1.00 (0.98–1.02)
* Climatic zone*					
Tropical or Subtropical [61, 65, 69, 70]	4; 2154	NA	NA	NA	0.73 (0.42–1.26)
Temperate [28, 29, 31–35, 39–42, 44, 45, 49, 50, 52, 54, 56–60, 62, 64]	24; 19,498	0.94 (0.89–0.99)	0.92 (0.85–0.99)	0.93 (0.87–0.99)	0.96 (0.92–1.00)
* Summer*					
Summer-inclusive [32, 34, 40, 42, 49, 52, 54, 56–59, 62, 64, 65, 69, 70]	16; 17,788	1.01 (0.96–1.06)	1.01 (0.94–1.08)	1.00 (0.94–1.07)	0.98 (0.95–1.02)
Summer-sparing [28, 29, 31, 33, 35, 39, 41, 44, 45, 50, 60, 61]	12; 3864	0.80 (0.71–0.91)	0.74 (0.65–0.89)	0.78 (0.66–0.92)	0.85 (0.73–1.00)
* Winter*					
Winter-dominant [28, 29, 31, 35, 39, 44, 45, 60]	8; 1658	0.69 (0.58–0.82)	0.70 (0.59–0.82)	0.80 (0.69–0.93)	0.79 (0.68–0.92)
Winter-non-dominant [32–34, 40–42, 49, 50, 52, 54, 56–59, 61, 62, 64, 65, 69, 70]	20; 19,994	0.99 (0.95–1.04)	0.99 (0.93–1.05)	0.99 (0.93–1.05)	0.98 (0.94–1.02)
**Sensitivity analysis**					
* Type of ARIs*					
Mixed upper and lower respiratory tract infections [29, 32–35, 42, 49, 54, 56, 60, 62, 64, 65]	13; 15,552	0.91 (0.82–1.02)	0.90 (0.79–1.02)	0.93 (0.84–1.02)	0.95 (0.91–1.00)
Upper respiratory tract infections [28, 39–41, 45, 50, 52, 54, 57–59, 61]	12; 5154	0.93 (0.86–1.00)	0.90 (0.80–1.00)	0.90 (0.79–1.01)	0.97 (0.90–1.04)
Lower respiratory tract infections [54]	1; 300	NA	NA	NA	0.94 (0.79–1.12)
Influenza [31, 44, 52, 58, 59, 61, 69]	7; 3425	0.92 (0.74–1.15)	0.89 (0.65–1.23)	0.91 (0.67–1.24)	0.96 (0.83–1.11)

*ARI* Acute respiratory infection, *NA* Not available

**Table 5:** Sensitivity analysis for bolus or monthly administration of vitamin D

Group	Study number; Patient number	Dose–response meta-analysis, RR (95% CI)			Pairwise Meta-analysis, RR (95% CI)
		400 IU/d	800 IU/d	1200 IU/d	
**Sensitivity analysis**					
Bolus or monthly administration [30, 36–38, 43, 46–48, 51, 53, 55, 63, 66–68]	15; 27,668	0.99 (0.96–1.03)	0.99 (0.94–1.05)	0.99 (0.93–1.06)	1.00 (0.98–1.03)
**Subgroups**					
* Age group (years)*					
< 7 [30, 37, 51, 67]	4; 4133	NA	NA	NA	0.99 (0.85–1.16)
7–17 [55]	1; 62	NA	NA	NA	1.01 (0.99–1.03)
18–65 [38, 46, 47]	3; 812	NA	NA	NA	0.99 (0.95–1.03)
> 65 [36, 43, 48, 53, 63, 66, 68]	7; 22,661	0.99 (0.97–1.00)	0.99 (0.95–1.01)	0.98 (0.94–1.01)	0.99 (0.97–1.02)
* Gender proportion (%)*					
Male > 60 [36, 47, 51, 67]	4; 1056	NA	NA	NA	1.08 (1.03–1.13)
Male ≤ 60 [30, 37, 38, 43, 46, 48, 53, 55, 63, 66, 68]	11; 26,612	0.99 (0.98–1.00)	0.99 (0.97–1.00)	0.98 (0.96–1.00)	0.99 (0.97–1.02)
* Comorbidity*					
General [37, 38, 43, 48, 53, 63, 66, 68]	8; 25,847	0.99 (0.98–1.01)	0.99 (0.96–1.01)	0.98 (0.95–1.01)	0.99 (0.98–1.01)
Disease-specific [30, 36, 46, 47, 51, 55, 67]	7; 1821	0.96 (0.87–1.07)	0.95 (0.83–1.09)	0.9 (0.84–1.08)	0.99 (0.93–1.06)
* Baseline 25-hydroxyvitamin D levels (nmol/L)*					
< 50 [36, 43, 46–48, 55]	6; 1209	0.96 (0.91–1.01)	0.93 (0.85–1.03)	0.92 (0.82–1.03)	1.01 (0.99–1.03)
> 50 [38, 53, 63, 66]	4; 6326	NA	NA	NA	1.00 (0.97–1.04)
* Trial duration (months)*					
< 4 [30]	1; 453	NA	NA	NA	0.77 (0.63–0.94)
4–12 [51, 67]	2; 634	NA	NA	NA	1.08 (1.04–1.13)
> 12 [36–38, 43, 46–48, 53, 55, 63, 66, 68]	12; 26,581	0.99 (0.98–1.00)	0.99 (0.97–1.00)	0.98 (0.96–1.00)	1.00 (0.99–1.01)
* Climatic zone*					
Tropical or Subtropical [67]	1; 310	NA	NA	NA	1.08 (1.04–1.13)
Temperate [30, 36–38, 43, 46–48, 51, 53, 55, 63, 66, 68]	14; 27,358	0.99 (0.98–1.00)	0.98 (0.97–1.00)	0.98 (0.96–1.00)	1.00 (0.98–1.02)
* Summer*					
Summer-inclusive [36–38, 43, 46–48, 51, 53, 55, 63, 66–68]	14; 27,215	1.00 (0.98–1.03)	1.00 (0.96–1.06)	1.01 (0.96–1.07)	1.01 (0.99–1.03)
Summer-sparing [30]	1; 453	NA	NA	NA	0.77 (0.63–0.94)
* Winter*					
Winter-dominant [30]	1; 453	NA	NA	NA	0.77 (0.63–0.94)
Winter-non-dominant [36–38, 43, 46–48, 51, 53, 55, 63, 66–68]	14; 27,215	1.00 (0.98–1.03)	1.01 (0.96–1.06)	1.01 (0.96–1.07)	1.01 (0.99–1.03
**Sensitivity analysis**					
* Type of ARIs*					
Mixed upper and lower respiratory tract infections [48, 53, 55, 63, 66, 67]	6; 6422	1.14 (0.00-NA)	1.25 (0.00-NA)	1.29 (0.00-NA)	1.03 (0.99–1.07)
Upper respiratory tract infections [38, 43, 46, 47, 68]	5; 17,241	0.99 (0.97–1.01)	0.98 (0.95–1.02)	0.98 (0.94–1.02)	0.98 (0.97–1.00)
Lower respiratory tract infections [30, 36, 37, 51]	4; 4005	NA	NA	NA	0.96 (0.79–1.16)
Influenza [53, 55]	2; 169	NA	NA	NA	0.89 (0.61–1.29)

*ARI* Acute respiratory infection, *NA* Not available

### Assessment of small-study effects

**Fig. 4:**
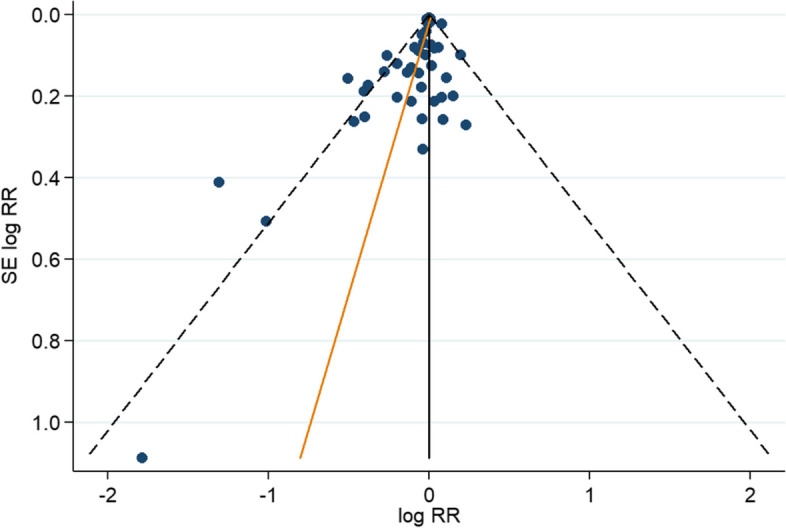
Funnel plot for assessment of overall small-study effects. Each dot represents an included study, located according to the logarithm of RR (X axis) and SE of logarithm of RR (Y axis). The dash black lines indicate the triangular region within which 95% of studies are expected to lie in the absence of biases. The plot asymmetry analysis was performed by Egger’s test, which suggests presence of small-study effects (*p* = 0.003). RR: relative risk; SE: standard error

The funnel plot of the included studies showed asymmetry, suggesting the potential presence of small-study effects (Fig. 4) (Egger’s test, *p* = 0.003).

## Discussion

### Main findings

The main dose–response meta-analysis revealed a J-shaped curve in the relationship between vitamin D supplementation dose and the preventive effects. The subgroup dose–response meta-analysis suggested that the optimal vitamin D supplementation doses were 400–1200 IU/d if taken in spring, autumn, and winter. Despite the absence of significant preventive effects observed in the main pairwise meta-analysis, subgroup pairwise meta-analysis suggested preventive effects were more evident in the subgroups of the daily dosing regimen, trial duration < 4 months, summer-sparing seasons, and winter-dominant seasons.

### Comparisons with previous meta-analyses

Previous meta-analyses have reported inconsistent findings regarding the preventive effects of vitamin D supplementation against ARIs [10–14]. Our main pairwise meta-analysis showed no significant preventive effects for supplemental vitamin D against ARIs (RR 0.99, 95% CI: 0.97–1.01, *I*^*2*^ = 49.6%, *p*_*het*_ < 0.001). Significant clinical and statistical between-study heterogeneity may lead to inconsistent preventive effects for vitamin D supplementation. The clinical heterogeneity may be attributed to several factors that may influence the effects of vitamin D supplementation, such as the dosing strategy. Martineau et al. [11] revealed that the subgroup using doses less than 800 IU/d showed a significant preventive effect of vitamin D supplementation (adjusted odds ratio: 0.80, 95% CI: 0.68–0.94, 5 studies) and Jolliffe et al. [12] noted that doses of vitamin D supplementation at 400–1000 IU/d exerted a preventive effect (RR: 0.70, 95% CI: 0.55–0.89, 10 studies).

As shown in Fig. 1, among the 43 trials, seven trials did not simply compare vitamin D supplementation with placebo. It could be difficult for pairwise meta-analysis to select adequate comparators for synthesizing the data, which might partly explain the inconsistent results in previous meta-analyses [10–12]. Furthermore, to combine several levels of vitamin D doses in a category, homogeneity of preventive effects within the same category must be assumed, which might not be adequate [71]. Finally, splitting studies into several dose categories may lead to lower power and precision [71] and not allow exploration of different types of dose–response relationships. For these reasons, we decided to treat vitamin D dose as a continuous variable, applying a dose–response meta-analysis [72, 73].

### Interpretation of current results

The current one‐stage model was able to better estimate the nonlinear dose–response curve based on aggregated data [74]. Because one‐stage model did not assume a particular type for the relationship, nonlinear relations could be investigated and applied to examine the fitness between the dose–response shape and data. Since the optimal dose and the dose–response relationship were unknown for vitamin D supplementation to prevent ARI, a data-driven approach rather than a pre-specified assumption may be justified for free examination. The results of the dose–response meta-analysis indicated that the restricted cubic spline fitted the data best, revealing a J-shaped relationship between the vitamin D supplementation dose and the preventive effects against ARI. The J-shaped relationship may be reasonable because epidemiological data [75] had also indicated a reverse J-shaped association between serum 25-hydroxyvitamin D concentration and all-cause mortality risk, with higher mortality noted at the two ends of the J-shaped curve. Therefore, the Institute of Medicine of the United States recommended avoiding serum 25-hydroxyvitamin D levels above 125 to 150 nmol/L [76]. A previous meta-analysis [77] also indicated that vitamin D supplementation doses of 3200–4000 IU/d were associated with an increased risk of adverse events. The preventive benefits of the supplemental vitamin D might not be linearly proportional to the intake amount. Nevertheless, the main dose–response meta-analysis did not identify preventive effects at pre-specified vitamin D supplementation doses (Table 2, Fig. 2).

Acknowledging that one size may not fit all, we explored the preventive effects in different subgroups. Interestingly, the subgroup dose–response meta-analysis indicated that the vitamin D supplementation dose at 400–1200 IU/d may be optimal for preventing ARIs in the summer-sparing and winter-dominant subgroups, i.e. during autumn, winter, and spring. Martineau et al. [11] and Jolliffe et al. [12] meta-analyses indicated that the preventive effects of vitamin D supplementation were observed at doses less than 800 IU/d and 400–1000 IU/d, respectively. The slightly inconsistent results between Martineau et al. [11] and Jolliffe et al. [12] may be caused by the seasonal effects, as noted in our study. The subgroup pairwise meta-analysis further indicated significant preventive effects of vitamin D supplementation in the subgroups of daily dosing regimen and trial duration < 4 months, consistent with previous meta-analyses [11, 12, 14]. Also, among the summer-sparing and winter-dominant subgroups, vitamin D supplementation demonstrated significant preventive effects against ARIs. This seasonal variation in the effects of vitamin D supplementation has not been reported in previous studies. Furthermore, in the winter-dominant subgroup, the statistical heterogeneity substantially decreased (*I*^*2*^:9.7%, Supplemental Fig. 9) compared with the main analysis (*I*^*2*^:49.6%, Fig. 3).

Taken together, the subgroup analysis suggests that in order to prevent ARIs, optimal intake of vitamin D is between 400–1200 IU daily for less than four months during spring, autumn or winter. The observation that supplemental vitamin D appears more effective in studies with summer-sparing or winter-dominant conditions has not been examined in previous meta-analyses [10–14]. It is important to emphasize that RCTs involving nutrients like vitamin D fundamentally differ from those involving drugs [78]. Specifically, for vitamin D, it is biologically impractical for the placebo group to have zero exposure to vitamin D. This means that comparisons in vitamin D RCTs always involve a placebo group that has some level of vitamin D exposure against an intervention group with a higher level of exposure. Vitamin D is mainly produced from precursors within the skin when exposed to ultraviolet-B light [79], which may lead to decreased 25-hydroxyvitamin D levels during winter due to reduced sunlight exposure [80]. These decreased baseline 25-hydroxyvitamin D levels may explain why vitamin D supplementation was most effective against ARIs during spring, autumn, or winter, as noted in the subgroup analysis. However, the preventive effects of vitamin D were not observed in the subgroup analysis of studies including participants with baseline 25-hydroxyvitamin D concentrations less than 50 nmol/L or conducted in temperate zones. Consequently, future RCTs should consider the starting 25-hydroxyvitamin D levels of participants and the concentrations of vitamin D reached after supplementation to clarify the effects of vitamin D supplementation.

### Future directions

Regarding the preventive effects of vitamin D against ARIs, the current study represents the most updated systematic review and meta-analysis since the COVID-19 pandemic. It incorporated one study [70] examining the effects of supplemental vitamin D in preventing COVID-19 among frontline healthcare workers. Furthermore, through dose–response meta-analysis, a J-shaped association between the vitamin D supplementation dose and its preventive effects was demonstrated for the first time, identifying an optimal daily supplemental vitamin D dose of 400–1200 IU. Subgroup analysis revealed that seasonal effects might play a significant role in the preventive efficacy of vitamin D. These study results may be pivotal in designing future RCTs. Since the onset of the COVID-19 pandemic, there has been increasing interest in supplementing vitamin D to improve outcomes [81]. With the evolution of mutant strains of SARS-CoV-2, further trials are warranted to investigate the preventive effects of vitamin D supplementation against COVID-19 and other ARIs.

### Study limitations

First, the present study employed data at the study level rather than the individual participant level. Meta-analysis of individual participant data may be performed in the future to investigate whether there is seasonal variation in the preventive effects of vitamin D supplementation. Second, most trials were conducted in high-income areas with a temperate climate. The generalization of our results to other areas may need more trials to support. Third, although we did not use any restrictions during the literature search, the funnel plot still indicated potential presence of small-study effects. Trials with a small sample size that demonstrated a potential increase in ARIs in vitamin D supplementation groups may be less likely to be published. Therefore, caution should be used in interpreting the study results because of the potential overestimated preventive effects of vitamin D supplementation. Fourth, the categorization for season-based subgroups was arbitrary. We examined the seasonal effects through two approaches and obtained similar conclusions, which may justify the classification based on the season. Fifth, the severity of ARIs was not considered in the analysis. Future research should investigate whether vitamin D supplementation can prevent severe morbidity or mortality associated with ARIs. Finally, the significant results noted in the subgroup analyses may have been caused by chances because of the increased number of subgroups tested. Nonetheless, the classification of subgroups was pre-specified, based on previous meta-analyses [10–14], rather than a data-driven approach. Despite this, the results of the subgroup analysis should be considered hypothesis-generating rather than definite conclusions.

## Conclusions

The dose–response meta-analysis revealed a J-shaped relationship between vitamin D supplementation dose and preventive effects against ARI. Vitamin D supplementation was noted to be more effective in the subgroups with daily dosing regimens or with trial durations < 4 months. Furthermore, seasonal variation was noted in the preventive effects of vitamin D supplementation, which suggested that the preventive effects of vitamin D supplementation may be more evident during spring, autumn, and winter at doses between 400 and 1200 IU/d.

### Supplementary Information

Additional file 1: Supplemental Table 1. Search strategies for each database. Supplemental Table 2. Accessory information of the included studies. Supplemental Table 3. Risk of bias assessment. Supplemental Figure 1. Dose-response and pairwise meta-analysis in the subgroup analysis stratified by age groups. Supplemental Figure 2. Dose-response and pairwise meta-analysis in the subgroup analysis stratified by male proportions. Supplemental Figure 3. Dose-response and pairwise meta-analysis in the subgroup analysis stratified by comorbidities. Supplemental Figure 4. Dose-response and pairwise meta-analysis in the subgroup analysis stratified by baseline 25-hydroxyvitamin D levels. Supplemental Figure 5. Dose-response and pairwise meta-analysis in the subgroup analysis stratified by dosing frequency. Supplemental Figure 6. Dose-response and pairwise meta-analysis in the subgroup analysis stratified by trial duration. Supplemental Figure 7. Dose-response and pairwise meta-analysis in the subgroup analysis stratified by climatic zone. Supplemental Figure 8. Dose-response and pairwise meta-analysis in the subgroup analysis stratified by summer. Supplemental Figure 9. Dose-response and pairwise meta-analysis in the subgroup analysis stratified by winter. Supplemental Figure 10. Dose-response and pairwise meta-analysis in the sensitivity analysis stratified by ARI definitions.

## Data Availability

No datasets were generated or analysed during the current study.

## Abbreviations

ARI: Acute respiratory infection
PRISMA: Preferred Reporting Items for Systematic Reviews and Meta-Analyses
RCT: Randomized controlled trial
RR: Relative risk
