# Supplementary material for: Optimal methods of vitamin D supplementation to prevent acute respiratory infections: a systematic review, dose–response and pairwise meta-analysis of randomized controlled trials
Source: Nutr J. 2024 Aug 14;23:92. doi: 10.1186/s12937-024-00990-w (PMC11323636; doi:10.1186/s12937-024-00990-w)
Supplement: Supplementary file 1 — Additional file 1: Supplemental Table 1. Search strategies for each database. Supplemental Table 2. Accessory information of the included studies. Supplemental Table 3. Risk of bias assessment. Supplemental Figure 1. Dose-response and pairwise meta-analysis in the subgroup analysis stratified by age groups. Supplemental Figure 2. Dose-response and pairwise meta-analysis in the subgroup analysis stratified by male proportions. Supplemental Figure 3. Dose-response and pairwise meta-analysis in the subgroup analysis stratified by comorbidities. Supplemental Figure 4. Dose-response and pairwise meta-analysis in the subgroup analysis stratified by baseline 25-hydroxyvitamin D levels. Supplemental Figure 5. Dose-response and pairwise meta-analysis in the subgroup analysis stratified by dosing frequency. Supplemental Figure 6. Dose-response and pairwise meta-analysis in the subgroup analysis stratified by trial duration. Supplemental Figure 7. Dose-response and pairwise meta-analysis in the subgroup analysis stratified by climatic zone. Supplemental Figure 8. Dose-response and pairwise meta-analysis in the subgroup analysis stratified by summer. Supplemental Figure 9. Dose-response and pairwise meta-analysis in the subgroup analysis stratified by winter. Supplemental Figure 10. Dose-response and pairwise meta-analysis in the sensitivity analysis stratified by ARI definitions. [file 12937_2024_990_MOESM1_ESM.docx]

**Supplemental Table 1**. Search strategies for each database

| Database | Search strategy |
| --- | --- |
| PubMed | #1 Vitamin D OR vitamin D2 OR vitamin D3 OR cholecalciferol OR ergocalciferol OR alphacalcidol OR alfacalcidol OR calcitriol OR paricalcitol OR doxercalciferol  #2 Acute Respiratory Infection OR Upper Respiratory Infection OR Lower Respiratory Infection OR Respiratory Tract Infection OR Common Cold OR Sinusitis OR Pharyngitis OR Laryngitis OR Laryngotracheobronchitis OR Tonsillitis OR peritonsillar abscess OR Croup OR Epiglottitis OR supraglottitis OR Otitis Media OR Pneumonia OR Bronchopneumonia OR Bronchitis OR Pleurisy OR Pleuritis  #3 (((COVID) OR (Coronavirus Disease)) OR (SARS-CoV-2)) OR (Severe acute respiratory syndrome coronavirus 2)  #4 (Acute Respiratory Infection OR Upper Respiratory Infection OR Lower Respiratory Infection OR Respiratory Tract Infection OR Common Cold OR Sinusitis OR Pharyngitis OR Laryngitis OR Laryngotracheobronchitis OR Tonsillitis OR peritonsillar abscess OR Croup OR Epiglottitis OR supraglottitis OR Otitis Media OR Pneumonia OR Bronchopneumonia OR Bronchitis OR Pleurisy OR Pleuritis) OR ((((COVID) OR (Coronavirus Disease)) OR (SARS-CoV-2)) OR (Severe acute respiratory syndrome coronavirus 2))  #5 (Vitamin D OR vitamin D2 OR vitamin D3 OR cholecalciferol OR ergocalciferol OR alphacalcidol OR alfacalcidol OR calcitriol OR paricalcitol OR doxercalciferol) AND ((Acute Respiratory Infection OR Upper Respiratory Infection OR Lower Respiratory Infection OR Respiratory Tract Infection OR Common Cold OR Sinusitis OR Pharyngitis OR Laryngitis OR Laryngotracheobronchitis OR Tonsillitis OR peritonsillar abscess OR Croup OR Epiglottitis OR supraglottitis OR Otitis Media OR Pneumonia OR Bronchopneumonia OR Bronchitis OR Pleurisy OR Pleuritis) OR ((((COVID) OR (Coronavirus Disease)) OR (SARS-CoV-2)) OR (Severe acute respiratory syndrome coronavirus 2))) |
| Embase | #1 vitamin AND d OR (vitamin AND d2) OR (vitamin AND d3) OR cholecalciferol OR ergocalciferol OR alphacalcidol OR alfacalcidol OR calcitriol OR paricalcitol OR doxercalciferol  #2 acute AND respiratory AND infection OR (upper AND respiratory AND infection) OR (lower AND respiratory AND infection) OR (respiratory AND tract AND infection) OR (common AND cold) OR sinusitis OR pharyngitis OR laryngitis OR laryngotracheobronchitis OR tonsillitis OR (peritonsillar AND abscess) OR croup OR epiglottitis OR supraglottitis OR (otitis AND media) OR pneumonia OR bronchopneumonia OR bronchitis OR pleurisy OR pleuritis  #3 covid OR (coronavirus AND disease) OR 'sars cov 2' OR (severe AND acute AND respiratory AND syndrome AND coronavirus AND 2)  #4 #2 OR #3  #5 #1 AND #4 |
| Cochrane Central Register of Controlled Trials | #1 Vitamin D OR vitamin D2 OR vitamin D3 OR cholecalciferol OR ergocalciferol OR alphacalcidol OR alfacalcidol OR calcitriol OR paricalcitol OR doxerocalciferol  #2 Acute Respiratory Infection OR Upper Respiratory Infection OR Lower Respiratory Infection OR Respiratory Tract Infection OR Common Cold OR Sinusitis OR Pharyngitis OR Laryngitis OR Laryngotracheobronchitis OR Tonsillitis OR peritonsillar abscess OR Croup OR Epiglottitis OR supraglottitis OR Otitis Media OR Pneumonia OR Bronchopneumonia OR Bronchitis OR Pleurisy OR Pleuritis  #3 covid OR (coronavirus AND disease) OR 'sars cov 2' OR (severe AND acute AND respiratory AND syndrome AND coronavirus AND 2)  #4 #2 OR #3  #5 #1 AND #4 |
| Web of Science | #1 (Vitamin D OR vitamin D2 OR vitamin D3 OR cholecalciferol OR ergocalciferol OR alphacalcidol OR alfacalcidol OR calcitriol OR paricalcitol OR doxercalciferol)  #2 ( Acute Respiratory Infection OR Upper Respiratory Infection OR Lower Respiratory Infection OR Respiratory Tract Infection OR Common Cold OR Sinusitis OR Pharyngitis OR Laryngitis OR Laryngotracheobronchitis OR Tonsillitis OR peritonsillar abscess OR Cr oup OR Epiglottitis OR supraglottitis OR Otitis Media OR Pneumonia OR Bronchopneumonia OR Bronchitis OR Pleurisy OR Pleuritis)  #3 ((COVID) OR (Coronavirus Disease)) OR (SARS-CoV-2)) OR (Severe acute respiratory syndrome coronavirus 2))  #4 #2 OR #3  #5 #1 AND #4 |
| ClinicalTrials.gov registry | Vitamin D AND ((respiratory AND infection) OR COVID) |

**Supplemental Table 2**. Accessory information of the included studies

| Author name, year, country | Excluded comorbidities that affect 25(OH)D metabolism ⧫ | 25(OH)D assay; EQA scheme | Adverse effects related to 25(OH)D administration ✤ |
| --- | --- | --- | --- |
| Li-Ng et al, 2009, USA[28] | Yes | RIA (DiaSorin); DEQAS | No adverse effects registered |
| Laaski et al,2010, Finland[29] | Yes | EIA (IDS); OCTEIA | No adverse effects registered |
| Manaseki-Holland et al, 2010, Afghanistan[30] | No | NA | No adverse effects registered |
| Urashima et al, 2010, Japan[31] | Yes | NA | No adverse effects registered |
| Kumar et al, 2011, India[32] | Yes | NR | No adverse effects registered |
| Majak et al, 2011, Poland[33] | Yes | RIA (BioSource); RIQAS | NR |
| Bergman et al, 2012, Sweden[34] | Yes | CLA (DiaSorin); DEQAS | No adverse effects registered |
| Camargo et al, 2012, Mongolia[35] | NR | LC-MS/MS; DEQAS | No adverse effects registered |
| Lehouck et al, 2012, Belgium[36] | Yes | RIA (DiaSorin); DEQAS | 4 cases of hypercalcemia |
| Manaseki- Holland et al, 2012, Afghanistan [37] | No | NR | 2 cases of hypercalcemia |
| Murdoch et al, 2012, New Zealand[38] | Yes | LC-MS/MS; DEQAS | No adverse effects registered |
| Marchisio et al, 2013, Italy [39] | No | CLA (DiaSorin) | No adverse effects registered |
| Rees et al, 2013, USA[40] | Yes | RIA (IDS OCTEIA) DEQAS | NR |
| Goodall et al, 2014, Canada[41] | Yes | NA | NR |
| Grant et al, 2014, New Zealand[42] | No | LC-MS/MS; DEQAS | NR |
| Tran et al, 2014, Australia [43] | Yes | CLA (DiaSorin); DEQAS | No adverse effects registered |
| Urashima et al, 2014, Japan[44] | Yes | NA | No adverse effects registered |
| Dubnov-Raz et al, 2015, Israel[45] | No | RIA (DiaSorin), DEQAS | NR |
| Martineau et al, 2015, ViDiAs Trial, England[46] | Yes | LC-MS/MS; DEQAS | No adverse effects registered |
| Martineau et al, 2015, ViDiCO Trial, England[47] | No | LC-MS/MS; DEQAS | No adverse effects registered |
| Martineau et al, 2015, ViDiFlu Trial, England [48] | Yes | LC-MS/MS; DEQAS | No adverse effects registered |
| Simpson et al, 2015, Australia[49] | Yes | LC-MS/MS; DEQAS | No adverse effects registered |
| Denlinger et al, 2016, USA [50] | NA | CLA (DiaSorin); VDSP | NR |
| Gupta et al, 2016, India [51] | Yes | RIA – Immunotech; SA / DiaSorin – EQA | No adverse effects registered |
| Aglipay et al, 2017, Canada[52] | No | CLA (Roche Elecsys); EQA scheme NR | No adverse effects registered |
| Ginde et al, 2017, USA[53] | Yes | LC-MS/MS; VDSP | No adverse effects registered |
| Hibbs et al, 2018, USA[54] | Yes | RIA (supplier NR); EQA scheme NR | No adverse effects registered |
| Lee et al, 2018, USA[55] | No | LC-MS/MS; DEQAS | No adverse effects registered |
| Rosendhal et al, 2018, Finland [56] | No | CLA (IDS-iSYS); VDSP | No adverse effects registered |
| Shimizu et al, 2018, Japan[57] | Yes | RIA (DiaSorin); EQA scheme NR | No adverse effects registered |
| Aloia et al, 2019, USA[58] | No | LC-MS/MS; NIST | No adverse effects registered |
| Arihiro et al, 2019, Japan[59] | No | RIA (DiaSorin); EQA scheme NR | No adverse effects registered |
| Hauger et al, 2019, Denmark[60] | NR | LC-MS/MS; DEQAS | NR |
| Loeb et al, 2019, Vietnam[61] | Yes | CLA (DiaSorin); DEQAS | No adverse effects registered |
| Bischop-Ferrari et al, 2020, Switzerland, France, Austria, Germany, Portugal[62] | Yes | LC-MS/MS; DEQAS | 19 cases of hypercalcemia; 15 cases of nephrocalcinosis |
| Camargo et al, 2020, New Zealand [63] | Yes | LC-MS (ABSciex API 4000), DEQAS | No adverse effects registered |
| Ganmaa et al, 2020, Mongolia[64] | No | EIA - BioMerieux DEQAS | No adverse effects registered |
| Mandlik et al, 2020, India[65] | Yes | EIA - DLD diagnostics scheme NR | NR |
| Rake et al, 2020, England[66] | Yes | CLA (Cobas 6000, Roche), scheme NR | No adverse effects registered |
| Jadhav et al, 2021, India[67] | Yes | NA | NR |
| Pham et al, 2020, Australia[68] | Yes | LC-MS; VDS | No adverse effects registered |
| Huang et al, 2022, Taiwan [69] | No | NA | No adverse effects registered |
| Villasis-Keever et al, 2022, Mexico [70] | Yes | LC-MS; (NR) | No adverse effects registered |
| 25(OH)D = 25-hydroxyvitamin D; NA: not applicable; NR: not reported  ⧫ Comorbidities which can affect 25(OH)D metabolism: obesity (body mass index > 30 kg/m^2); current tobacco use; malabsorption, malignancies, current liver or kidney disorders which reduces the organ functionality, use of medications that interfere with vitamin D metabolism (drugs activating the Pregnane-X-receptor, such as: Phenytoin, Carbamazepine, Cyclophosphamide, Taxol, Tamoxifen, Clotrimazole, Rifampicin, etc.).  ✤Adverse effect related to 25(OH)D administration: hypercalcemia, hypervitaminosis D, hypercalciuria, nephrocalcinosis, kidney stones (other symptoms related to hypercalcemia have not been included due to the lack of specificity). | | | |

**Supplemental Table 3**. Risk of bias assessment

| **Author name, publication year** | **Randomization process** | **Deviations from intended interventions** | **Missing outcome data** | **Measurement of the outcome** | **Selection of the reported result** | **Overall Bias** |
| --- | --- | --- | --- | --- | --- | --- |
| Li-Ng et al, 2009 [28] | Low | Low | Low | Low | Low | Low |
| Laaski et al, 2010 [29] | Low | Low | Some concerns: High percentage of missing outcome data | Low | Low | Some concerns |
| Manaseki-Holland et al, 2010 [30] | Low | Low | Low | Low | Low | Low |
| Urashima et al, 2010 [31] | Low | Low | Low | Low | Low | Low |
| Kumar et al, 2011 [32] | Low | Low | Low | Low | Low | Low |
| Majak et al, 2011 [33] | Low | Low | Low | Low | Low | Low |
| Bergman et al, 2012 [34] | Low | Low | Low | Low | Low | Low |
| Camargo et al, 2012 [35] | Low | Low | Low | Low | Low | Low |
| Lehouck et al, 2012 [36] | Low | Low | Low | Low | Low | Low |
| Manaseki-Holland et al, 2012 [37] | Low | Low | Low | Low | Low | Low |
| Murdoch et al, 2012 [38] | Low | Low | Low | Low | Low | Low |
| Marchisio et al, 2013 [39] | Low | Low | Low | Low | Low | Low |
| Rees et al, 2013 [40] | Low | Low | Low | Low | Low | Low |
| Goodall et al, 2014 [41] | Low | Low | Low | Low | Low | Low |
| Grant et al, 2014 [42] | Low | Low | Low | Low | Low | Low |
| Tran et al, 2014 [43] | Low | Low | Low | Low | Low | Low |
| Urashima et al, 2014 [44] | Low | Low | Low | Low | Low | Low |
| Dubnov-Raz et al, 2015 [45] | Low | Low | Some concerns: High percentage of missing outcome data | Low | Low | Some concerns |
| Martineau et al, 2015, ViDiAs Trial, [46] | Low | Low | Low | Low | Low | Low |
| Martineau et al, 2015, ViDiCO Trial, [47] | Low | Low | Low | Low | Low | Low |
| Martineau et al, 2015, ViDiFlu Trial [48] | Low | Low | Low | Low | Low | Low |
| Simpson et al, 2015 [49] | Low | Low | Low | Low | Low | Low |
| Denlinger et al, 2016 [50] | Low | Low | Low | Low | Low | Low |
| Gupta et al, 2016 [51] | Low | Low | Low | Low | Low | Low |
| Aglipay et al, 2017 [52] | Low | Low | Low | Low | Low | Low |
| Ginde et al, 2017 [53] | Low | Low | Low | Low | Low | Low |
| Hibbs et al, 2018 [54] | Low | Low | Low | Low | Low | Low |
| Lee et al, 2018 [55] | Low | Low | Low | Low | Low | Low |
| Rosendhal et al, 2018 [56] | Low | Low | Low | Low | Low | Low |
| Shimizu et al, 2018 [57] | Low | Low | Low | Low | Low | Low |
| Aloia et al, 2019 [58] | Low | Low | Low | Low | Low | Low |
| Arihiro et al, 2019 [59] | Low | Low | Low | Low | Low | Low |
| Hauger et al, 2019 [60] | Low | Low | Low | Low | Low | Low |
| Loeb et al, 2019 [61] | Low | Low | Low | Low | Low | Low |
| Bischop-Ferrari et al, 2020 [62] | Low | Low | Low | Low | Low | Low |
| Camargo et al, 2020 [63] | Low | Low | Low | Low | Low | Low |
| Ganmaa et al, 2020 [64] | Low | Low | Low | Low | Low | Low |
| Mandlik et al, 2020 [65] | Low | Low | Low | Low | Low | Low |
| Rake et al, 2020 [66] | Low | Low | Low | Low | Low | Low |
| Jadhav et al, 2021 [67] | Low | Low | Some concerns: Missing data were all in the placebo group | Low | Low | Some concerns |
| Pham et al, 2021 [68] | Low | Low | Low | Low | Low | Low |
| Huang et al, 2022 [69] | Low | Low | Low | Low | Low | Low |
| Villasis-Keever et al, 2022 [70] | Low | Low | Low | Low | Low | Low |

**Supplemental Figure 1. Dose-response and pairwise meta-analysis in the subgroup analysis stratified by age groups.**

**
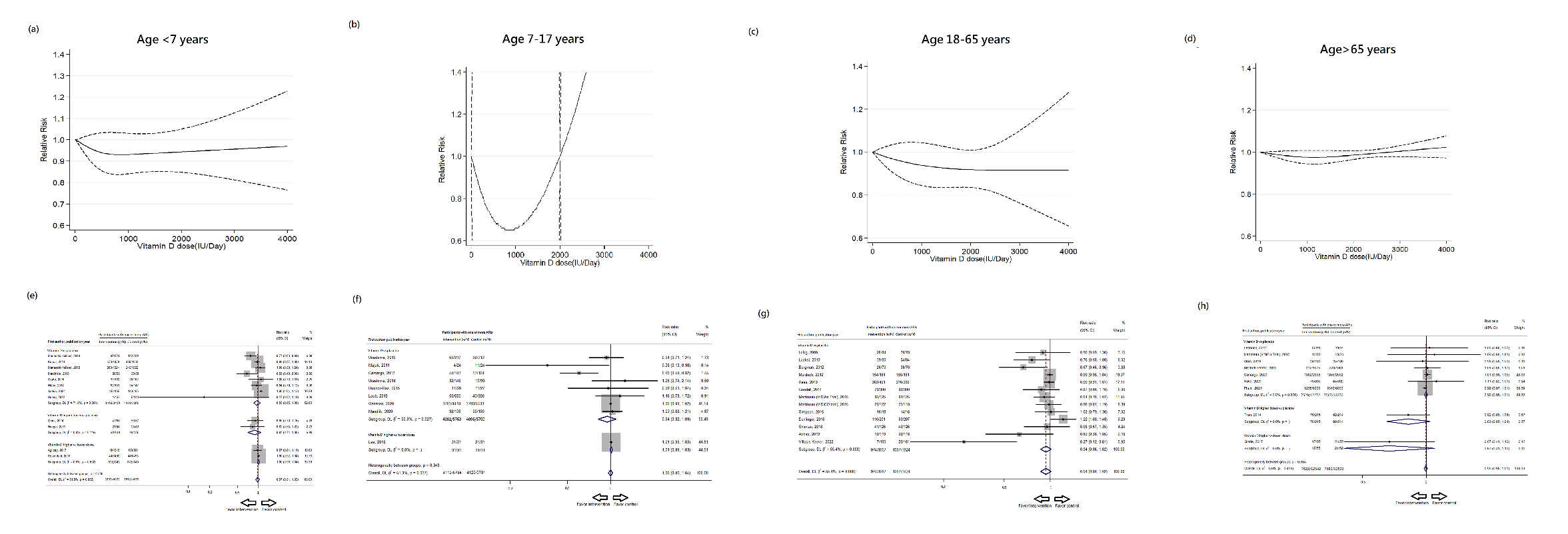
**

(a)-(d) J-shaped dose-response curves were noted in each subgroup, except for the subgroup of age 7-17 years, in which the convergence was not achieved. Dashed black lines are 95% point-wise CIs estimated by the respective 1-stage random-effects model. (e)-(h) Forest plots of the summary risk ratios comparing proportions of participants with one or more ARIs between intervention and control groups. In the comparison of vitamin D higher doses vs plabebo, there were two or more levels of vitamin D doses in each included study; only the group with highest vitamin D dose and the placebo in each study were selected for pooling. In the comparison of vitamin D higher vs lower doses, there were no placebo control group in included studies; the two groups with different vitamin D doses in each study were selected for pooling. CI: confidence interval; DL: DerSimonian and Laird random effects model; n: number of participants with one or more ARI; N:total number of participants in the study group.

**Supplemental Figure 2.** Dose-response and pairwise meta-analysis in the subgroup analysis stratified by male proportions.

**
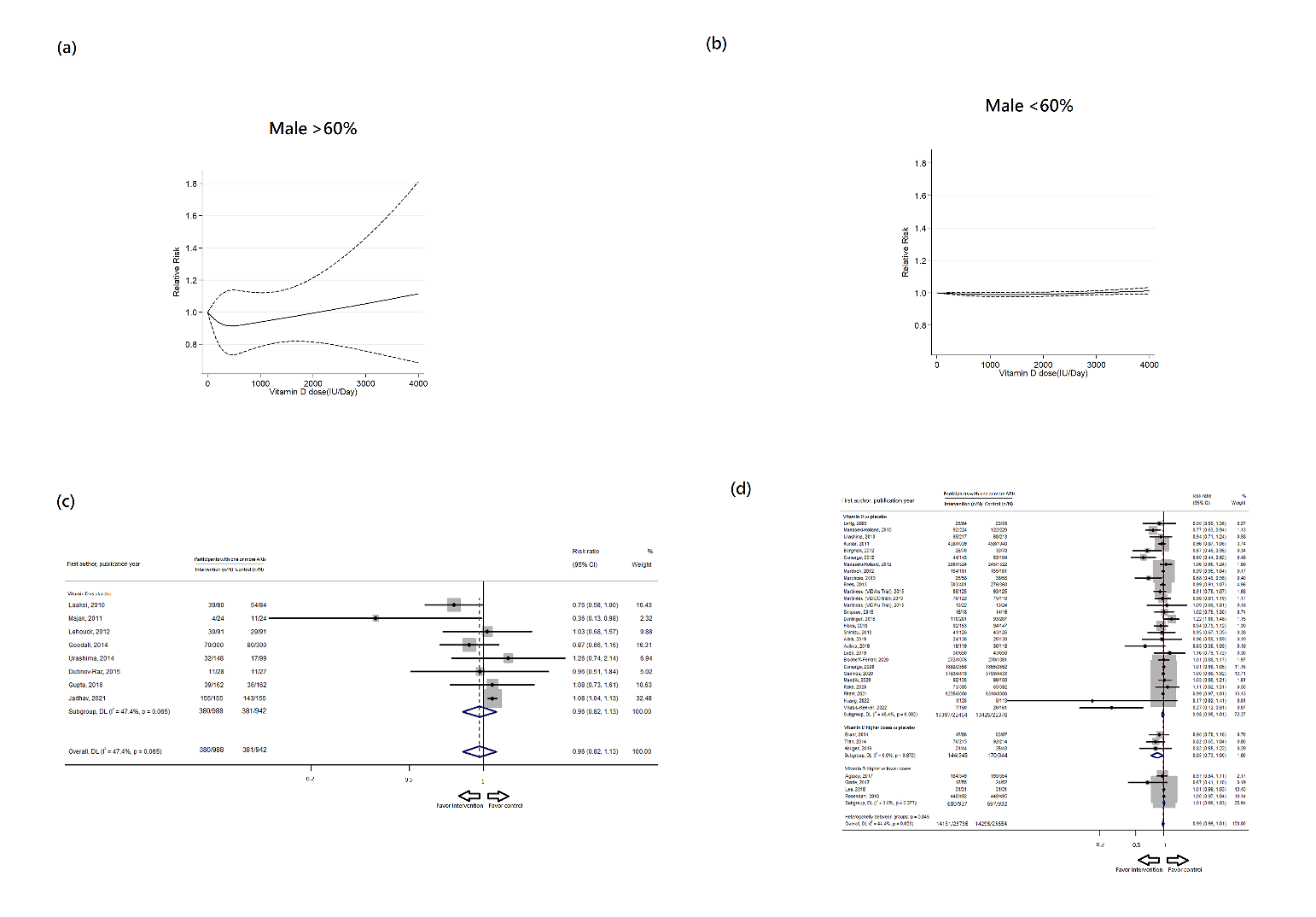
**

(a)(b) J-shaped dose-response curves were noted in each subgroup. Dashed black lines are 95% point-wise CIs estimated by the respective 1-stage random-effects model. (c)(d) Forest plots of the summary risk ratios comparing proportions of participants with one or more ARIs between intervention and control groups. In the comparison of vitamin D higher doses vs plabebo, there were two or more levels of vitamin D doses in each included study; only the group with highest vitamin D dose and the placebo in each study were selected for pooling. In the comparison of vitamin D higher vs lower doses, there were no placebo control group in included studies; the two groups with different vitamin D doses in each study were selected for pooling. CI: confidence interval; DL: DerSimonian and Laird random effects model; n: number of participants with one or more ARI; N:total number of participants in the study group.

**Supplemental Figure 3.** Dose-response and pairwise meta-analysis in the subgroup analysis stratified by comorbidities.


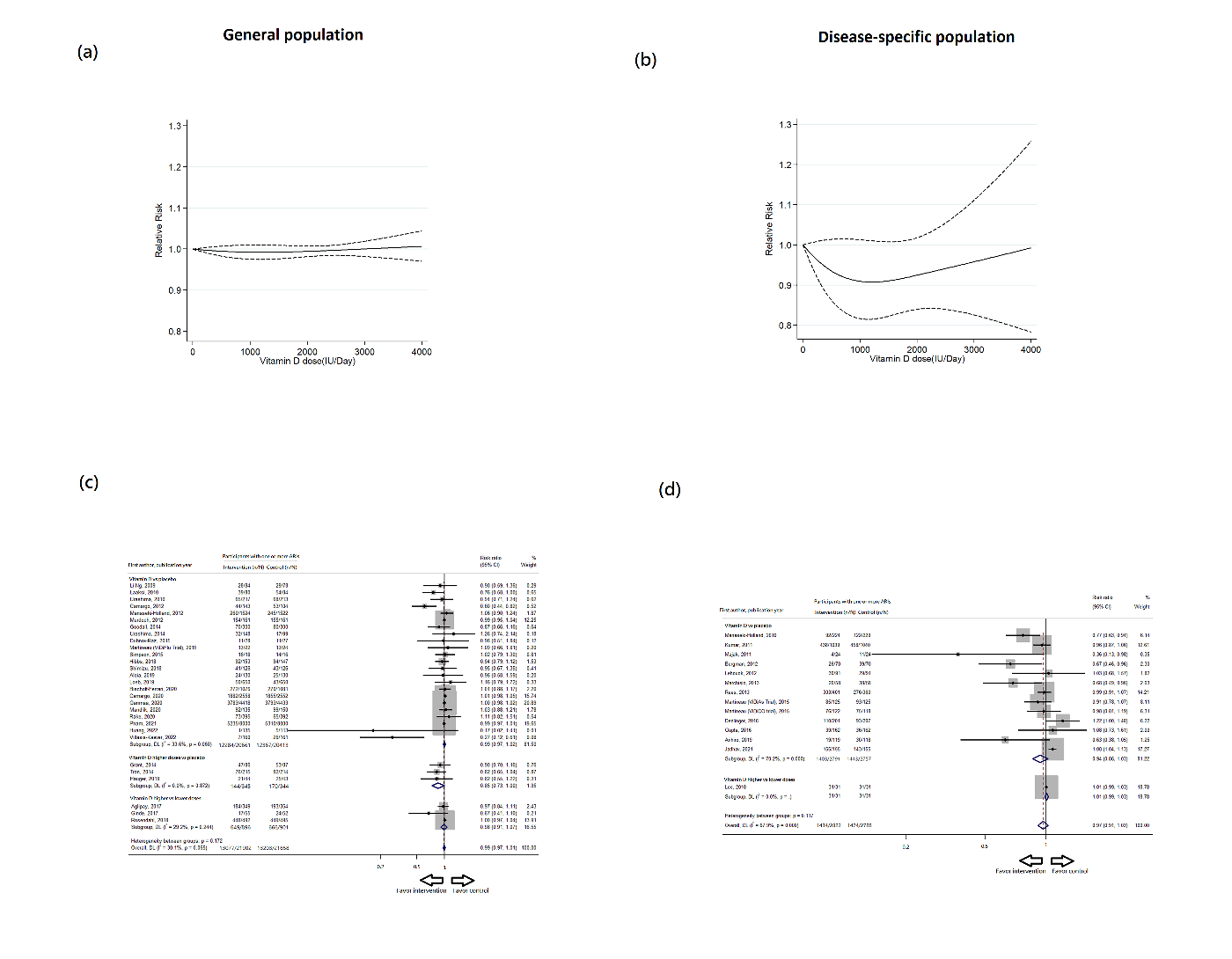


(a)(b) J-shaped dose-response curves were noted in each subgroup. Dashed black lines are 95% point-wise CIs estimated by the respective 1-stage random-effects model. (c)(d) Forest plots of the summary risk ratios comparing proportions of participants with one or more ARIs between intervention and control groups. In the comparison of vitamin D higher doses vs plabebo, there were two or more levels of vitamin D doses in each included study; only the group with highest vitamin D dose and the placebo in each study were selected for pooling. In the comparison of vitamin D higher vs lower doses, there were no placebo control group in included studies; the two groups with different vitamin D doses in each study were selected for pooling. CI: confidence interval; DL: DerSimonian and Laird random effects model; n: number of participants with one or more ARI; N:total number of participants in the study group.

**Supplemental Figure 4.** Dose-response and pairwise meta-analysis in the subgroup analysis stratified by baseline 25-hydroxyvitamin D levels.


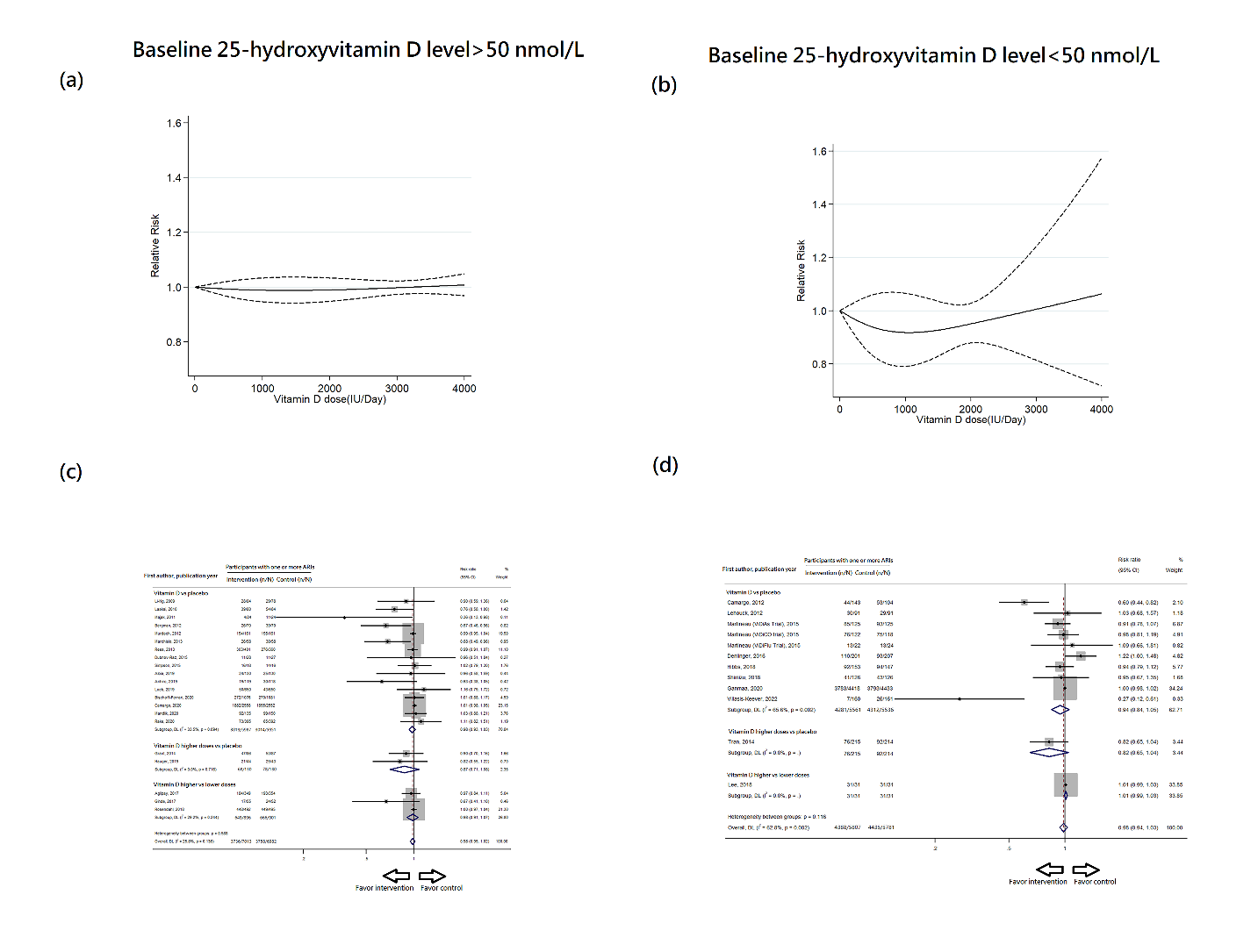


(a)(b) J-shaped dose-response curves were noted in each subgroup. Dashed black lines are 95% point-wise CIs estimated by the respective 1-stage random-effects model. (c)(d) Forest plots of the summary risk ratios comparing proportions of participants with one or more ARIs between intervention and control groups. In the comparison of vitamin D higher doses vs plabebo, there were two or more levels of vitamin D doses in each included study; only the group with highest vitamin D dose and the placebo in each study were selected for pooling. In the comparison of vitamin D higher vs lower doses, there were no placebo control group in included studies; the two groups with different vitamin D doses in each study were selected for pooling. CI: confidence interval; DL: DerSimonian and Laird random effects model; n: number of participants with one or more ARI; N:total number of participants in the study group.

**Supplemental Figure 5.** Dose-response and pairwise meta-analysis in the subgroup analysis stratified by dosing frequency.


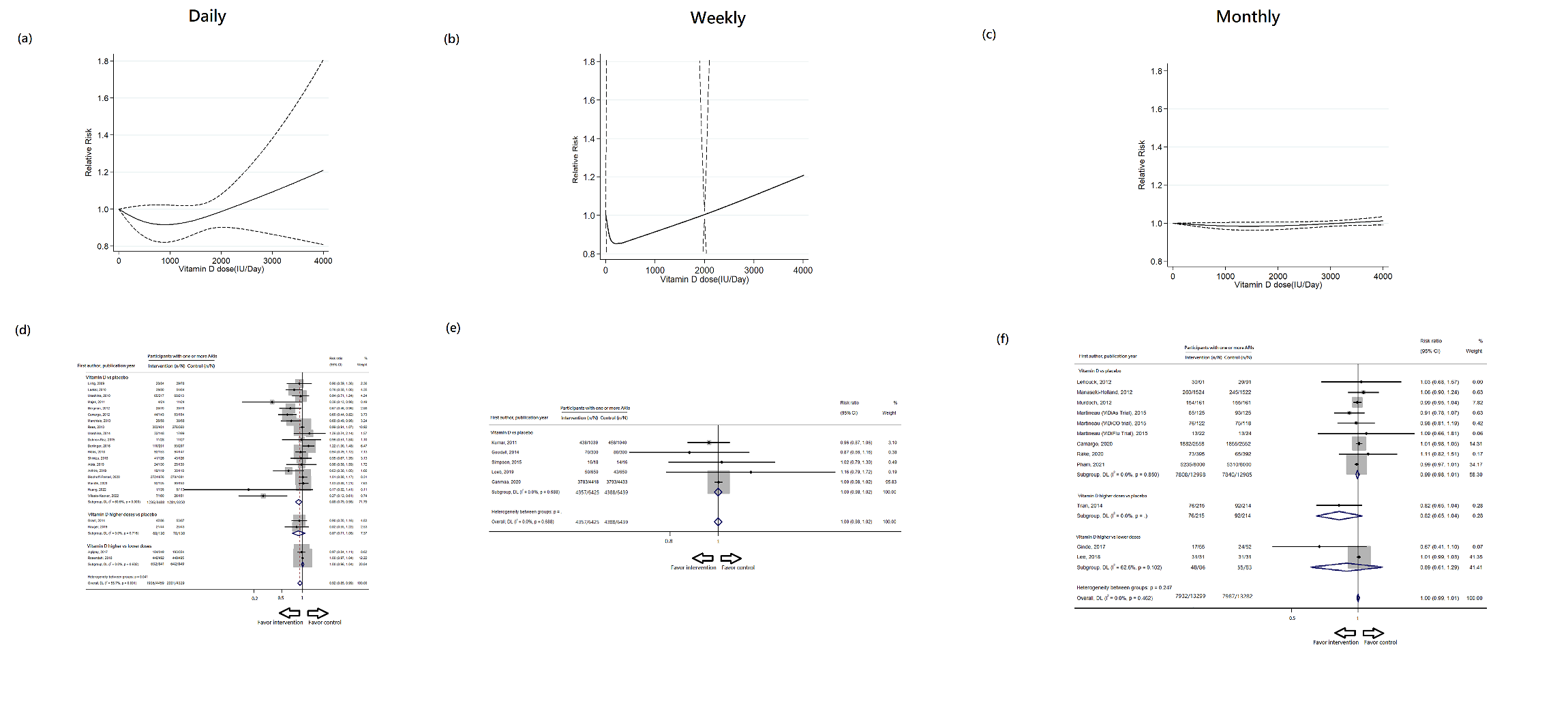


For the subgroup of bolus administration, meta-analysis was not performed because there were only three studies in this subgroup. (a)-(c) J-shaped dose-response curves were noted in each subgroup, except for the subgroup of weekly administration, in which the convergence was not achieved. Dashed black lines are 95% point-wise CIs estimated by the respective 1-stage random-effects model. (d)-(f) Forest plots of the summary risk ratios comparing proportions of participants with one or more ARIs between intervention and control groups. In the comparison of vitamin D higher doses vs plabebo, there were two or more levels of vitamin D doses in each included study; only the group with highest vitamin D dose and the placebo in each study were selected for pooling. In the comparison of vitamin D higher vs lower doses, there were no placebo control group in included studies; the two groups with different vitamin D doses in each study were selected for pooling. CI: confidence interval; DL: DerSimonian and Laird random effects model; n: number of participants with one or more ARI; N:total number of participants in the study group.

**Supplemental Figure 6.** Dose-response and pairwise meta-analysis in the subgroup analysis stratified by trial duration.


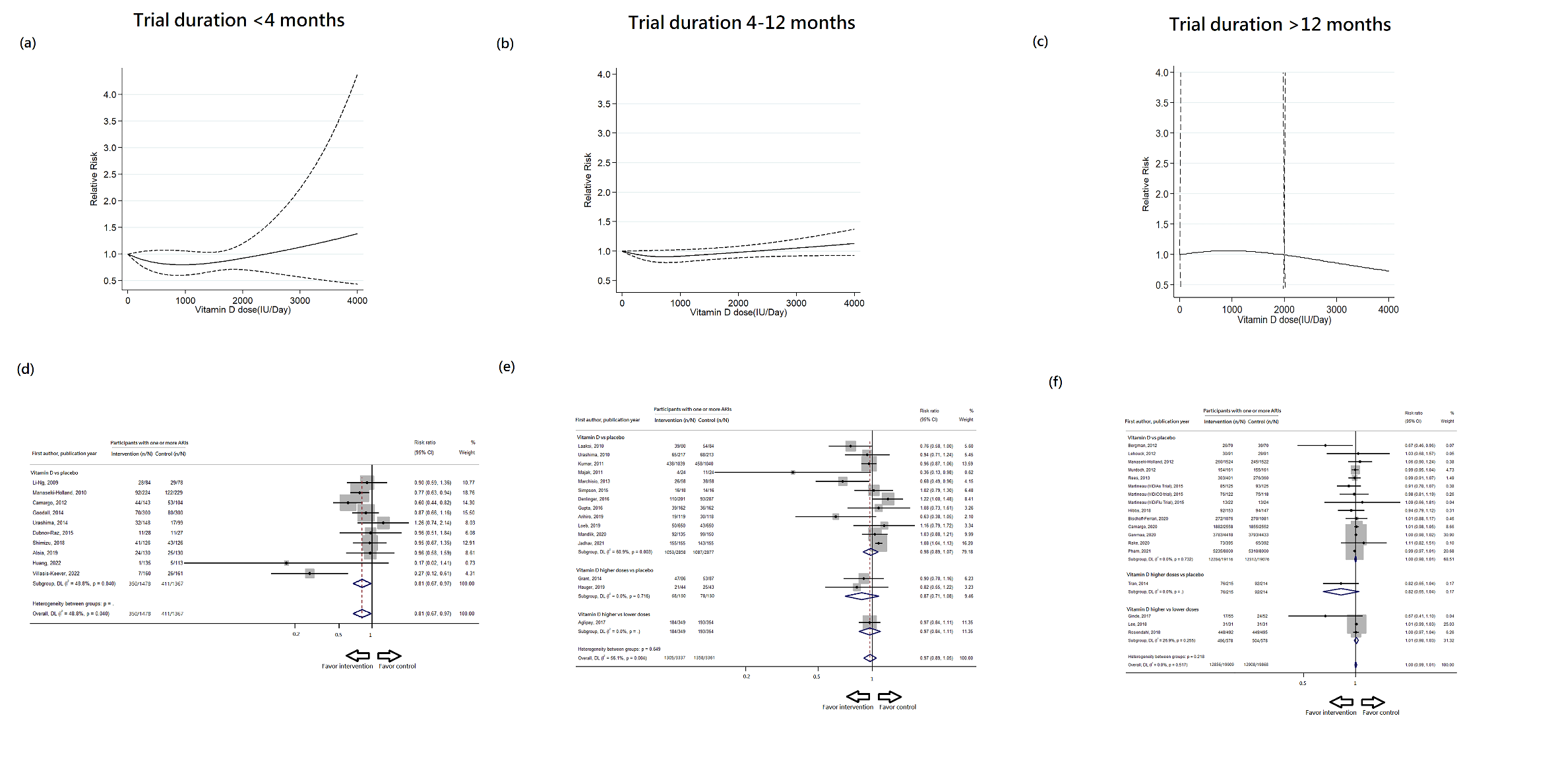


(a)-(c) J-shaped dose-response curves were noted in each subgroup. Dashed black lines are 95% point-wise CIs estimated by the respective 1-stage random-effects model. (d)-(f) Forest plots of the summary risk ratios comparing proportions of participants with one or more ARIs between intervention and control groups. In the comparison of vitamin D higher doses vs plabebo, there were two or more levels of vitamin D doses in each included study; only the group with highest vitamin D dose and the placebo in each study were selected for pooling. In the comparison of vitamin D higher vs lower doses, there were no placebo control group in included studies; the two groups with different vitamin D doses in each study were selected for pooling. CI: confidence interval; DL: DerSimonian and Laird random effects model; n: number of participants with one or more ARI; N:total number of participants in the study group.

**Supplemental Figure 7.** Dose-response and pairwise meta-analysis in the subgroup analysis stratified by climatic zone.


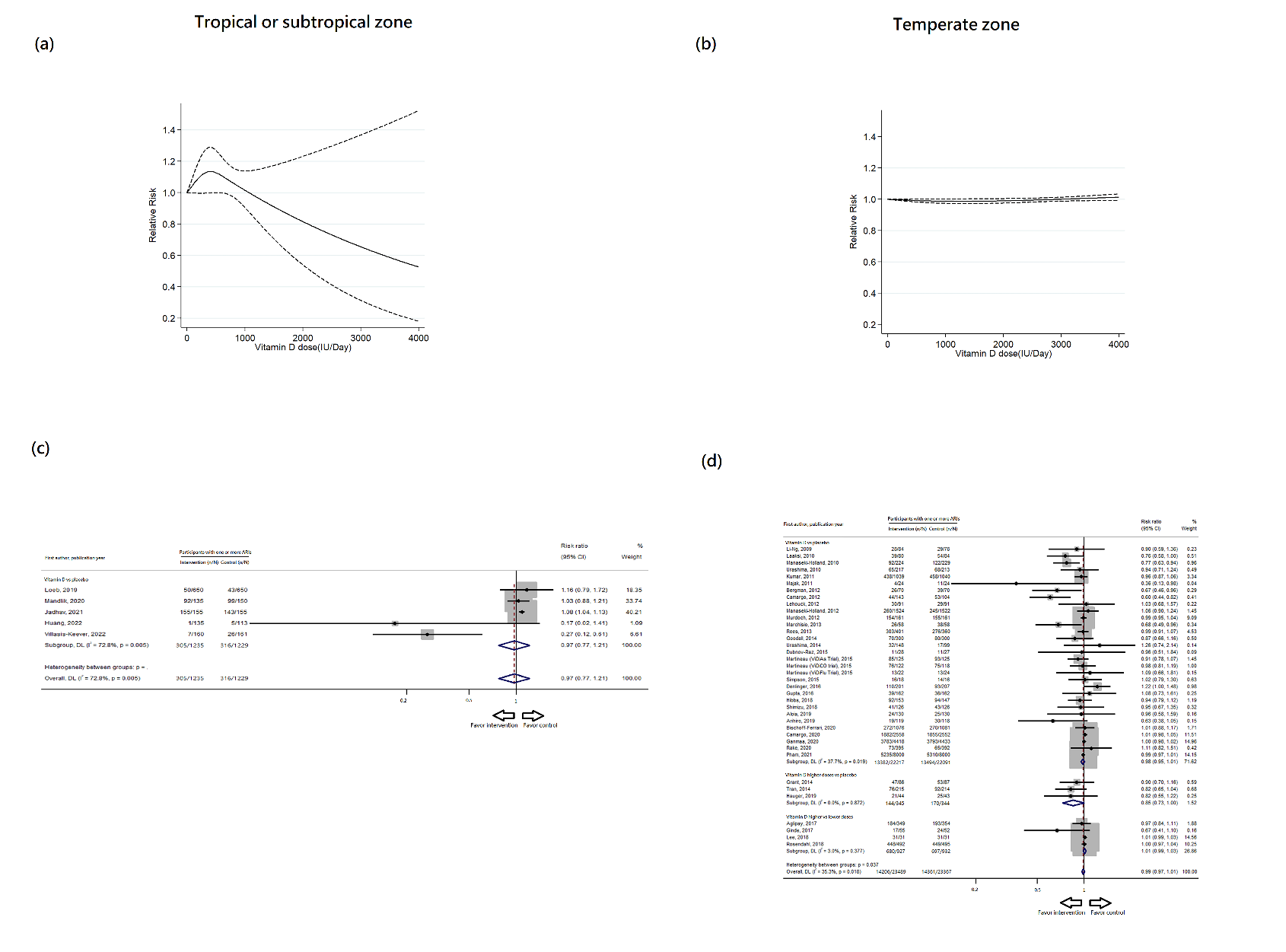


(a)(b) J-shaped dose-response curves were noted in each subgroup. Dashed black lines are 95% point-wise CIs estimated by the respective 1-stage random-effects model. (c)(d) Forest plots of the summary risk ratios comparing proportions of participants with one or more ARIs between intervention and control groups. In the comparison of vitamin D higher doses vs plabebo, there were two or more levels of vitamin D doses in each included study; only the group with highest vitamin D dose and the placebo in each study were selected for pooling. In the comparison of vitamin D higher vs lower doses, there were no placebo control group in included studies; the two groups with different vitamin D doses in each study were selected for pooling. CI: confidence interval; DL: DerSimonian and Laird random effects model; n: number of participants with one or more ARI; N:total number of participants in the study group.

**Supplemental Figure 8.** Dose-response and pairwise meta-analysis in the subgroup analysis stratified by summer.


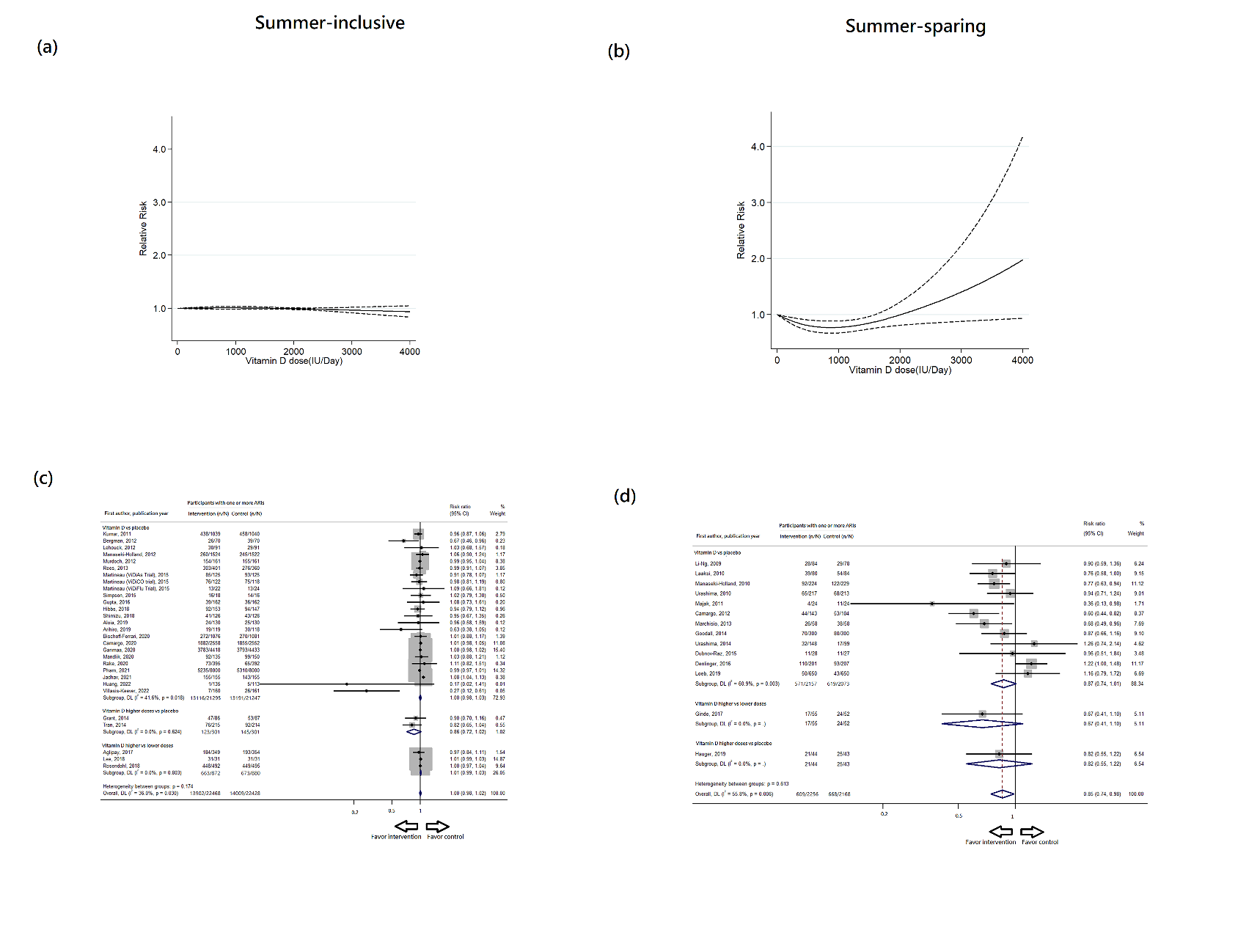


(a)(b) J-shaped dose-response curves were noted in each subgroup. Dashed black lines are 95% point-wise CIs estimated by the respective 1-stage random-effects model. The upper bound of the 95% confidence interval (dash lines) is noted to be below the horizontal line of relative risk of one at a certain range of vitamin D doses, indicating the presence of significant preventive effects of vitamin D supplementation at this range of doses. (c)(d) Forest plots of the summary risk ratios comparing proportions of participants with one or more ARIs between intervention and control groups. In the comparison of vitamin D higher doses vs plabebo, there were two or more levels of vitamin D doses in each included study; only the group with highest vitamin D dose and the placebo in each study were selected for pooling. In the comparison of vitamin D higher vs lower doses, there were no placebo control group in included studies; the two groups with different vitamin D doses in each study were selected for pooling. CI: confidence interval; DL: DerSimonian and Laird random effects model; n: number of participants with one or more ARI; N:total number of participants in the study group.

**Supplemental Figure 9.** Dose-response and pairwise meta-analysis in the subgroup analysis stratified by winter.


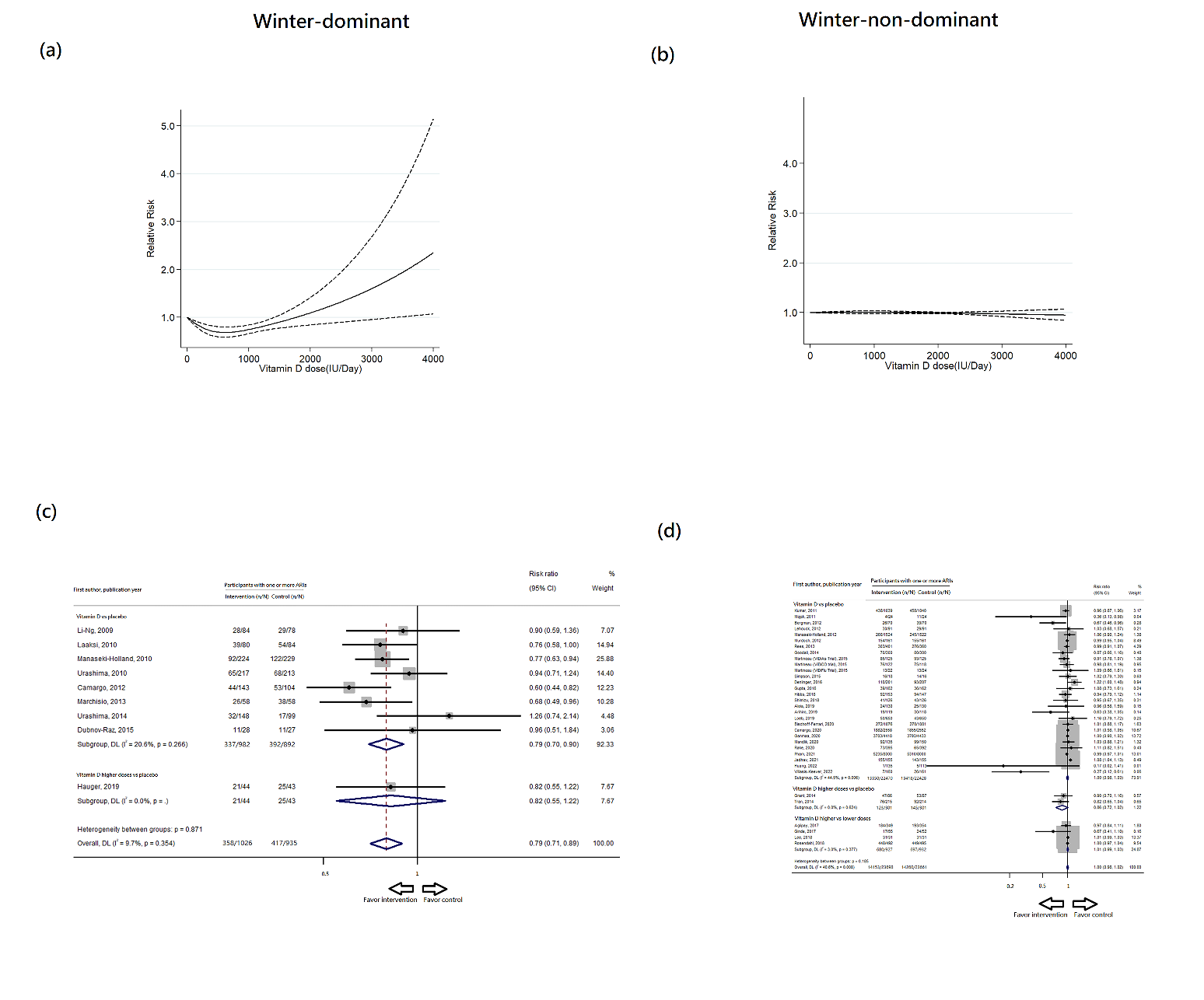


(a)(b) J-shaped dose-response curves were noted in each subgroup. Dashed black lines are 95% point-wise CIs estimated by the respective 1-stage random-effects model. The upper bound of the 95% confidence interval (dash lines) is noted to be below the horizontal line of relative risk of one at a certain range of vitamin D doses, indicating the presence of significant preventive effects of vitamin D supplementation at this range of doses. (c)(d) Forest plots of the summary risk ratios comparing proportions of participants with one or more ARIs between intervention and control groups. In the comparison of vitamin D higher doses vs plabebo, there were two or more levels of vitamin D doses in each included study; only the group with highest vitamin D dose and the placebo in each study were selected for pooling. In the comparison of vitamin D higher vs lower doses, there were no placebo control group in included studies; the two groups with different vitamin D doses in each study were selected for pooling. CI: confidence interval; DL: DerSimonian and Laird random effects model; n: number of participants with one or more ARI; N:total number of participants in the study group.

**Supplemental Figure 10.** Dose-response and pairwise meta-analysis in the sensitivity analysis stratified by ARI definitions.


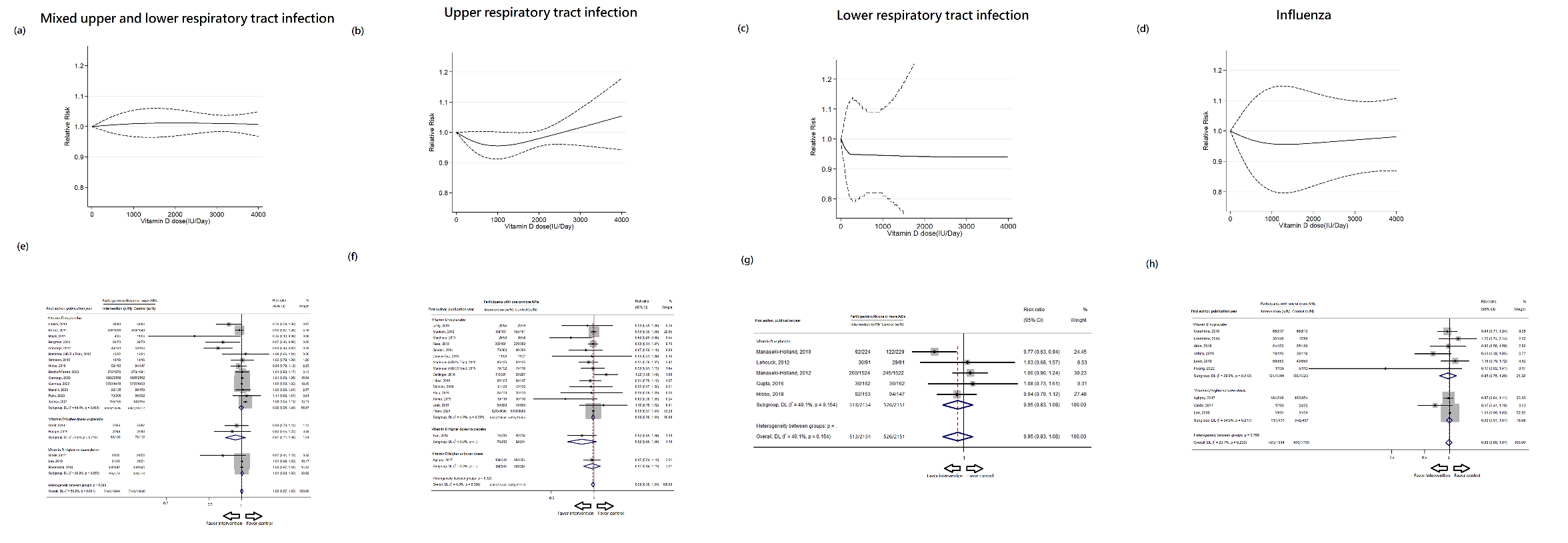


(a)-(d) J-shaped dose-response curves were noted in each subgroup. Dashed black lines are 95% point-wise CIs estimated by the respective 1-stage random-effects model. (e)-(h) Forest plots of the summary risk ratios comparing proportions of participants with one or more ARIs between intervention and control groups. In the comparison of vitamin D higher doses vs plabebo, there were two or more levels of vitamin D doses in each included study; only the group with highest vitamin D dose and the placebo in each study were selected for pooling. In the comparison of vitamin D higher vs lower doses, there were no placebo control group in included studies; the two groups with different vitamin D doses in each study were selected for pooling. CI: confidence interval; DL: DerSimonian and Laird random effects model; n: number of participants with one or more ARI; N:total number of participants in the study group.
